# Detection and Molecular Characterization of Adenoviruses in Captive and Free-Roaming African Green Monkeys (*Chlorocebus sabaeus*): Evidence for Possible Recombination and Cross-Species Transmission

**DOI:** 10.3390/v15071605

**Published:** 2023-07-22

**Authors:** Diana M. Mancuso, Kerry Gainor, Kerry M. Dore, Christa A. Gallagher, Katalina Cruz, Amy Beierschmitt, Yashpal S. Malik, Souvik Ghosh

**Affiliations:** 1 Department of Biomedical Sciences, Ross University School of Veterinary Medicine, Basseterre P.O. Box 334, Saint Kitts and Nevis; dianamancuso@students.rossu.edu (D.M.M.); kerrygainor@students.rossu.edu (K.G.); kerrymdore@gmail.com (K.M.D.); cgallagher@rossvet.edu.kn (C.A.G.); kathacruz@gmail.com (K.C.); abeierschmitt@rossvet.edu.kn (A.B.); 2 National Coordinator, CABI/GEF/UNEP Regional Project—‘Preventing the COSTS of Invasive Alien Species in Barbados and OECS Countries’ in St. Kitts, Ministry of Environment, Climate Action and Constituency Development, Basseterre 00265, Saint Kitts and Nevis; 3 Behavioral Science Foundation, Estridge Estate, Basseterre P.O. Box 428, Saint Kitts and Nevis; 4 College of Animal Biotechnology, Guru Angad Dev Veterinary and Animal Science University, Ludhiana 141012, India; malikyps@gmail.com

**Keywords:** adenovirus, African green monkey (AGM), captive AGM, DNA-dependent DNA polymerase, free-roaming AGM, hexon, *Human mastadenovirus-F*, penton base, *Simian mastadenovirus-F*

## Abstract

In the present study, 31 samples (12 fecal, 9 nasal and 10 rectal swabs) from 28/92 (30.43%, 10 captive and 18 free-roaming African green monkeys (AGMs, *Chlorocebus sabaeus*)) apparently healthy AGMs in the Caribbean Island of St. Kitts tested positive for adenoviruses (AdVs) by DNA-dependent DNA polymerase (*pol*)-, or hexon-based screening PCR assays. Based on analysis of partial deduced amino acid sequences of Pol- and hexon- of nine AGM AdVs, at least two AdV genetic variants (group-I: seven AdVs with a *Simian mastadenovirus-F* (SAdV-F)/SAdV-18-like Pol and hexon, and group-II: two AdVs with a SAdV-F/SAdV-18-like Pol and a *Human mastadenovirus-F* (HAdV-F)/HAdV-40-like hexon) were identified, which was corroborated by analysis of the nearly complete putative Pol, complete hexon, and partial penton base sequences of a representative group-I (strain KNA-08975), and -II (KNA-S6) AdV. SAdV-F-like AdVs were reported for the first time in free-roaming non-human primates (NHPs) and after ~six decades from captive NHPs. Molecular characterization of KNA-S6 (and the other group-II AdV) indicated possible recombination and cross-species transmission events involving SAdV-F-like and HAdV-F-like viruses, corroborating the hypothesis that the evolutionary pathways of HAdVs and SAdVs are intermingled, complicated by recombination and inter-species transmission events, especially between related AdV species, such as HAdV-F and SAdV-F. To our knowledge, this is the first report on detection and molecular characterization of AdVs in AGMs.

## 1. Introduction

Adenoviruses (AdV), members of the family *Adenoviridae*, are ubiquitous viruses that have been reported in a wide variety of animal species (mammals, birds, reptiles, amphibians, and fish), and in environmental samples [1,2,3,4,5]. Although many of the AdVs are considered as low-grade pathogens that typically cause asymptomatic or subclinical infections, certain AdVs have been linked to acute clinical disease in humans and other animals, especially in immunocompetent hosts [1,2,3,4]. The AdV genome consists of a single linear molecule of double-stranded DNA that encode up to ~40 different viral polypeptides [1]. Among the major AdV proteins, the viral DNA-dependent DNA polymerase (Pol) is required for the replication of AdV genome [2,4,6]. Phylogenetic analysis of the Pol sequences constitutes an important basis for the taxonomic classification of AdVs [1]. The AdV capsid is primarily composed of hexon (major capsid antigen), penton base and fiber proteins that mediate attachment of AdVs to host cells [1,2,4,7]. Based on phylogenetic analysis, genomic organization, ‘G + C’ content of AdV genomes, and biological properties, AdVs have been classified into at least six genera (*Atadenovirus*, *Aviadenovirus*, *Ichtadenovirus*, *Mastadenovirus*, *Siadenovirus and Testadenovirus*) so far [1].

AdVs of non-human primates (NHP) (also referred to as simian AdVs (SAdVs)) belong to the genus *Mastadenovirus* (consists of only mammalian AdVs) [1]. To date, SAdVs have been reported in great apes (bonobo, chimpanzee and gorilla), Old World monkeys (OWM) (baboon, colobus monkey, black-crested mangabey, Campbell’s mona monkey, Diana monkey, golden snub-nosed monkey, grivet, macaque, mandrill and patas monkey), New World monkeys (NWM) (black howler, gray-bellied night monkey, marmoset, spider monkey, squirrel monkey, tamarin, titi monkey and tufted capuchin monkey) and prosimians (lemur) [1,3]. Simian AdVs have been detected in apparently healthy NHPs and in NHPs with various clinical conditions, such as diarrhea, pneumoenteritis, conjunctivitis, hepatitis, and pancreatitis [8,9,10,11,12,13]. Since SAdVs and human AdVs (HAdVs) are derived from related host species, they have been shown to possess highly similar genomes that share common ancestry, and their complex evolutionary pathways appear to be intertwined, complicated by intra- and cross-species recombination events [3,14,15,16,17,18]. This has been reflected in the taxonomical classification of SAdVs, with the great ape AdVs belonging to mastadenovirus species *Human mastadenovirus*-A (HAdV-A), -B, -C, -E, or -F, and several OWM AdVs grouping with a single human-derived AdV within species HAdV-G [1,19]. On the other hand, mastadenovirus species *Simian mastadenovirus-A* (SAdV-A) to SAdV-I consist of SAdVs from OWMs, whilst the NWM SAdVs constitute a single mastadenovirus species, *Platyrrhini mastadenovirus A*, that is phylogenetically distinct from the clade of HAdVs and other SAdVs (from great apes and OWMs) [1,3,18].

The premise that SAdVs are genetically related to HAdVs, yet antigenically far enough to elude the pre-existing specific immunity in humans, has attracted interest in employing certain SAdV isolates as potential gene therapy/delivery vectors and as oncolytic agents in human medicine [20,21]. On the other hand, the intermingled phylogenetic clustering patterns of SAdVs and HAdVs underscore the significant zoonotic and anthroponotic potential of AdVs, which in conjunction with the (i) wide distribution of AdVs in NHP and human populations, (ii) increased exposure of humans to NHPs and vice versa due to anthropogenic events, and (iii) evidence for recombination events between HAdVs and SAdVs (that might result in emergence of novel, more virulent AdV strains) raises public health concerns [1,3,9,14,15,16,17,18,22]. To date, there are several examples, supported by genomic, serological, and/or clinical evidence, that indicate possible AdV cross-species transmission events between humans and NHPs, including a single instance of human-to-human transmission of NHP-derived AdVs [3,9,22,23,24,25].

The African green monkey (AGMs) (*Chlorocebus sabaeus*), members of OWMs, are native to West Africa, and were introduced in the Caribbean region during the 1600s [26,27]. The Caribbean Island of St. Kitts (surface area of 176 square km) is home to a large population of AGMs (Estimated to be about ~40,000, which equals to the local human population) [26,27,28,29]. The invasive AGM population has significantly impacted agricultural production, with economic implications, necessitating efforts to control their numbers in the island [26,27,28,29]. The St. Kitts AGMs enter human habitats and often come in close contact with humans, livestock, and companion animals, offering an ideal environment for cross-species transmission of pathogens, as evidenced from previous studies [30,31,32,33,34,35]. Although AdVs have been detected in a wide variety of OWMs [1,3], there is a lack of information on the molecular epidemiology of AdVs circulating in AGMs. In a recent study, all of the few fecal samples (*n* = 4) obtained from free-roaming AGMs in Senegal tested negative for AdVs by qPCR and conventional PCR assays [23]. Adenoviruses have been propagated in kidney cell lines derived from AGMs [36], and anti-AdV antibodies have been reported in AGMs [37]. In the present study, based on nasal and fecal/rectal samples from AGMs in St. Kitts, we report for the first-time detection and molecular characterization of AdVs in captive and free-roaming AGMs, providing evidence for possible recombination and cross-species transmission events.

## 2. Materials and Methods

### 2.1. Sampling

The present study was based on samples obtained from 92 apparently healthy AGMs on the island of St. Kitts, and included (i) rectal and nasal swabs from 52 free-roaming AGMs (sampled during September 2022–January 2023), (ii) fecal samples from 5 free-roaming AGMs (July 2014 and November 2014), (iii) 5 fecal samples from caged, pet AGMs (March 2015), and (iv) fecal samples from 30 captive AGMs (September 2015–December 2015) housed in individual cages at the quarantine facility of Behavioral Science Foundation (BSF), a fully accredited biomedical NHP research facility in St. Kitts.

Following trapping, the free-roaming AGMs were sedated with ketamine (dose 7–10 mg/kg, intramuscular injection in hindleg) (Ketamidor^®^, Chanelle Pharma, Galway, Ireland), and once the animals reached proper sedation levels, nasal and rectal samples were obtained using sterile swabs (MicroTest™ M4RT, Thermo Fisher Scientific Inc., Waltham, MA, USA) (one each for nasal and rectal sample) from respective orifices, and transferred into separate sterile tubes containing viral transport medium (MicroTest™ M4RT, Thermo Fisher Scientific Inc., Waltham, MA, USA). The fecal samples were obtained from the rectal orifice (using a lubricated sterile glove) of the sedated AGM and transported into a sterile container (Sigma-Aldrich, Milwaukee, WI, USA). Some of the free-roaming AGMs were sampled at the trapping site, whilst others were sampled immediately after transportation to the nearby BSF facility.

On the other hand, samples were obtained from the captive and pet AGMs by scooping a small volume of the material from the top of the fecal pile immediately after the NHP had defecated in the cage. The captive monkeys at BSF were sampled the next day, or a few days after they were trapped. All samples were preserved at −80 °C until further analysis. The procedures for trapping and sampling of AGMs were approved by the Ross University School of Veterinary Medicine (RUSVM), St. Kitts, IACUC 2014 #14-6-032 and IACUC #22-5-11 and followed the RUSVM IACUC approved “Monkey trapping policies and procedures” (SOP # NHP001, April 2022).

### 2.2. Amplification of AdV DNA

Viral DNA was extracted from the AGM samples using the QIAamp DNA Mini Kit (Qiagen Sciences, Germantown, MD, USA). The samples were screened for the presence of AdV DNA using a nested PCR assay that targeted a partial stretch of the AdV *pol* gene (~250 bp) as described by Roy et al. [38]. Samples that tested negative with the Roy primers were further screened for AdVs with a hexon-based PCR assay as reported by Ba’nyai et al. [8]. Primers used in PCR/semi-nested PCR/nested PCR assays to obtain the nearly complete Pol, complete hexon, and partial penton base coding sequences of the AGM AdVs were designed in the present study and are shown in Supplementary Table S1. The PCR assays were carried out using the Platinum™ Taq DNA Polymerase (Invitrogen™, Thermo Fisher Scientific Corporation, Waltham, MA, USA), or the QIAGEN Hot Star Taq Master Mix Kit (Qiagen Sciences, Germantown, MD, USA) according to the manufacturer’s instructions. To rule out contamination issues, sterile water was used as a negative control in all PCR reactions.

### 2.3. Nucleotide Sequencing

The Wizard^®^ SV Gel and PCR Clean-Up kit (Promega, Madison, WI, USA) was used to purify the PCR products following the protocol outlined by the manufacturer. Nucleotide (nt) sequences were obtained by the Sanger dideoxy chemistry using the ABI Prism Big Dye Terminator Cycle Sequencing Ready Reaction Kit (Applied Biosystems, Foster City, CA, USA) on an ABI 3730XL Genetic Analyzer (Applied Biosystems, Foster City, CA, USA). Nucleotide sequences were obtained in both directions.

### 2.4. Sequence Analysis

Homology search for related AdV sequences was performed using the standard BLASTN and BLASTP program (Basic Local Alignment Search Tool, www.ncbi.nlm.nih.gov/blast, accessed on 20 May 2023). The putative coding regions and corresponding deduced amino acid sequences (aa) were determined using the ORF finder (https://www.ncbi.nlm.nih.gov/orffinder/, accessed on 15 May 2023), and confirmed by BLASTN and BLASTP analysis, respectively. Pairwise nt and deduced aa sequence identities (%) were calculated using the BLASTN and BLASTP program, respectively, with the ‘align two or more sequences’ option (accessed 20 May 2023). Multiple alignments were carried out using the CLUSTALW program (https://www.genome.jp/tools-bin/clustalw, accessed on 22 May 2023) with default parameters. Phylogenetic analysis was performed by the maximum likelihood (ML) method using the MEGA11 software [39], with the LG+I+G model of substitution and 1000 bootstrap replicates, as described previously [1,3]. Recombination analysis was carried out using the RDP4 program [40], with parameters described previously [41]. The ‘G + C’ content (%) of AdV sequences were determined using the GC Content Calculator (https://www.biologicscorp.com/tools/GCContent/, accessed 20 May 2023).

### 2.5. GenBank Accession Numbers

The GenBank accession numbers for the AGM AdV sequences determined in this study are OR066890-OR066927.

## 3. Results

### 3.1. Detection of AdVs in AGMs

**Figure 1:**
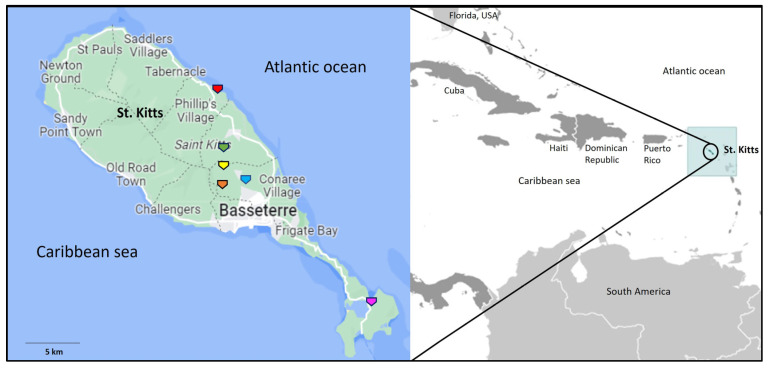
Map showing the geographical location of the Caribbean Island of St. Kitts (**Right**). The map was adapted from https://www.cia.gov/the-world-factbook/countries/saint-kitts-and-nevis/locator-map (accessed on 15 May 2023). Map of St. Kitts showing the locations where the African green monkeys were trapped/sampled (**Left**). The trapping site in Cedar grove, Fountain Mountain, Green hill, Monkey hill, and Southeastern peninsula is shown with an orange, yellow, green, blue, and pink pentagon arrow, respectively, whilst the BSF facility is indicated with a red pentagon arrow. The map of St. Kitts was adapted from https://www.google.com/maps (accessed on 15 May 2023).

The sampling/trapping sites for the captive (at the BSF quarantine facility) and free-roaming AGMs are shown in Figure 1. Since the pan-*pol* nested PCR assay described by Wellehan et al. [42] has been used to detect a wide variety of AdVs, especially novel AdVs in wildlife [43], a subset of AGM samples (*n* = 20) from the present study were initially screened using the Wellehan primers. Seven samples yielded the expected ~300 bp amplicon, which were shown to be amplification of non-AdV genomes by sequencing of the PCR products followed by BLASTN analysis. These observations, and similar findings from other studies (Gainor et al. [44], and 33 of 37 fecal/rectal samples from bats on St. Kitts tested false positive for AdVs) raise concerns on the specificity of the Wellehan primers in AdV screening assays.

In the present study, all the AGM samples from St. Kitts were screened by a *pol*-based nested PCR assay that has been used to detect AdVs in NHPs [23,25,38]. A total of 28 samples (9 fecal, 9 nasal and 10 rectal samples) tested positive for AdVs using the *pol* PCR assay. Furthermore, screening of the *pol* PCR negative samples with a hexon-based PCR assay [8] revealed the presence of AdVs in 3 additional fecal samples. All screening results were confirmed by sequencing (and BLASTN analysis) of the PCR amplicons. The details of the AGMs that tested positive for AdVs are shown in Table 1. Overall, 31 samples (12 fecal, 9 nasal and 10 rectal) from 28 AGMs (30.43% (*n* = 92), 3 animals tested positive for both nasal and rectal swabs) tested positive for the presence of AdV DNA. The rates of detection of AdVs in nasal, fecal, and rectal samples were 17.3% (9/52 AGMs), 30% (12/40), and 19.2% (10/52), respectively. The AdV detection rates in captive, free-roaming (trapped), and caged pet AGMs were 33.33% (10/30 AGMs), 31.57% (18/57), and 0% (0/5), respectively. Although the sample size from free-roaming AGMs was almost twice of that from captive AGMs, the AdV detection rates were comparable between both groups. Since the samples were obtained from captive AGMs in quarantine cages the next day, or a few days after trapping, it is more likely that they acquired the infection before introduction into the BSF facility. All the AdV positive AGMs were apparently healthy at the time of sampling.

### 3.2. Analysis of the Partial Pol and Hexon Sequences of AGM AdV Strains

By BLASTN analysis, the AdV partial *pol* sequences (*n* = 25, ~200 bp after trimming end sequences, from the *pol*-based screening PCR assay [38]) from AGMs shared ~ 98–100% nt identities between themselves, and 95.5–98.9% identities with cognate *pol* sequences of SAdV-F/SAdV-17 (*Mastadenovirus species*/AdV serotype, or genotype) strain B-105 (GenBank accession number KP329566) and SADV-F/SAdV-18 strain C676 (FJ025931) from grivet monkeys (*Chlorocebus aethiops*) [1,45,46,47,48,49], followed by identities of 84.54–88.83% with those of other AdVs. Although the remaining partial *pol* sequences (*n* = 3) and all the 3 AdV partial hexon sequences (~250 bp after trimming end sequences, from the hexon-based screening PCR assay [8]) shared maximum homology with SADV-F/SAdV-18, they lacked quality (phred value < 40) and were not considered for sequence identities. Between themselves, the *pol* of SAdV-F/SAdV-17 and -18 are highly conserved (97.4% nt sequence identities), whilst their hexon are genetically divergent (83.20% identities). Since the AGM AdV partial *pol* sequences were closely related to both SAdV-F/SAdV-17 and -18, 24 of the 31 AdV positive samples were subjected to a semi-nested PCR assay (designed in this study, Supplementary Table S1) targeting a partial stretch of the hexon (corresponding to nt 18458-nt 19307 of SAdV-F/SAdV-18) that is genetically divergent between SAdV-F/SAdV-17 and -18. Seven samples were not included, as they exhibited faint amplification with the *pol*-based screening PCR assay and were not available in sufficient volumes. Out of the 24 samples, 9 tested positive with the hexon-based semi-nested PCR assay, which included only one of the 3 samples that were positive for the hexon-based screening PCR assay. Surprisingly, the 13 *pol* PCR positive AdV samples, negative for the hexon-based semi-nested PCR assay, also tested negative with the hexon-based screening PCR assay. A flow chart summarizing the work pipeline and the outcomes from different PCR assays is shown in Supplementary Figure S1.

**Figure 2:**
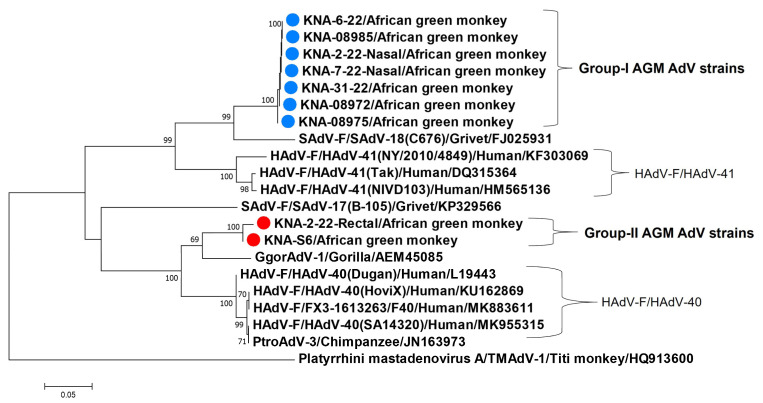
Phylogenetic analysis of the partial deduced amino acid (aa) sequences (~250 aa) of the putative hexons of group-I and -II (shown with blue and red circles, respectively) simian adenovirus (SAdVs) strains from African green monkeys (AGMs) in St. Kitts with those of *Human mastadenovirus-F* (human adenovirus-40 and -41), *Simian mastadenovirus-F* (SAdV-17 and -18), and SAdV strains GgorAdV-1 and PtroAdV-3. The name of the virus/host of detection are shown for the AGM SAdVs, whilst the *Mastadenovirus species*/name of the virus/host of detection/GenBank accession number have been mentioned for the HAdV-F and SAdV-F strains, and the name of the virus/host of detection/GenBank accession number for strains GgorAdV-1 and PtroAdV-3. *Platyrrhini mastadenovirus A*/TMAdV-1/Titi monkey/HQ913600 was used as the outgroup sequence. Bootstrap values <60% are not shown. Scale bar, 0.05 substitutions per aa residue.

Based on nt sequence identities, the partial hexon sequences (~750 bp after trimming end sequences, from hexon-based semi-nested PCR) of the nine AGM AdV strains were classified into two groups, consisting of seven (group-I) and two AdV (group-II) strains, respectively. Members within each group shared 100% nt sequence identities between themselves, whilst nt and deduced aa identities of ~67% and ~70%, respectively, were observed between the groups. Interestingly, a single AGM (KNA-2-22) tested positive for both group-I (nasal swab) and -II (rectal swab) viruses. The partial hexon aa sequences (~250 aa) of group-I viruses shared maximum identities of ~ 88% with cognate sequences of SAdV-F/SAdV-18 and <83% identities with other AdVs, whilst group-II viruses shared maximum identities of ~91% with AdV strain GgorAdV-1 (GenBank accession number AEM45085) from a gorilla (*Gorilla gorilla*) [19], followed by ~86% identities with AdV strain PtroAdV-3 (JN163973) from a chimpanzee (*Pan troglodytes*) [19] and some HAdV-F/HAdV-40 strains. These findings were corroborated by phylogenetic analysis of the partial hexon aa sequences (Figure 2). *Mastadenovirus* species HAdV-F (consisting of HAdV-40 and -41) and SAdV-F (SAdV-17 and -18) are considered as sister taxons [17], whilst SAdV strains GgorAdV-1 and PtroAdV-3 were shown to be closely related to HAdV-F [19], and strain PtroAdV-3 was proposed to be transmitted from humans to a chimpanzee [19]. Of note here, a third SAdV strain, ROC2012 (MN136539, from a moustached guenon, *Cercopithecus cephus*) was also shown to cluster with HAdV-F AdVs [50]. However, only a 275 nt hexon sequence was available for ROC2012 in the GenBank database, which was not sufficient for analysis.

Taken together, the partial Pol sequences of group-I and -II AGM AdV strains were closely related to both SAdV-17 and -18, whilst the partial hexon sequences of group-I and -II viruses were more related to those of SAdV-F/SAdV-18 and HAdV-F/HAdV-40 (and SAdVs from a gorilla and a chimpanzee that were closely related to HAdV-F), respectively, than those of other AdVs (Figure 2). Considering these observations, two AGM AdV strains, KNA-S6 and KNA-08975, representing group-I and -II viruses, respectively, and available in sufficient volumes, were selected for further molecular characterization.

### 3.3. Analysis of the Nearly Complete Pol, Complete Hexon and Partial Penton Coding Sequences of AGM AdV Strains

The nearly complete Pol (3534 nt, corresponding to nt 5241-nt 8789 of SAdV-F/SAdV-18), complete hexon (2778 nt and 2769 nt for KNA-S6 and KNA-08975, respectively), and partial penton base (902 nt, 3′-region of penton base, corresponding to nt 14423-nt 15322 of SAdV-F/SAdV-18) coding sequences of AGM AdV strains KNA-S6 and KNA-08975 were obtained using primers shown in Supplementary Table S1. On the other hand, repeated attempts to amplify the 5′-region of the penton base with 4 different forward primers (Supplementary Table S1) and 2 reverse primers (designed from the obtained penton base sequences of KNA-S6 and KNA-08975) in separate PCR assays failed. Since SAdV strains GgorAdV-1 and PtroAdV-3 (related to HAdV-F) have been characterized for partial genomes [19], they were excluded from further analysis. In the present study, the analyses of the putative Pol, hexon and penton base of KNA-S6 and KNA-08975 were based on deduced aa sequences, as reported in previous studies [1,3,16], and the outcomes were similar to those observed with nt sequences. To rule out biases, phylogenetic analyses were performed with both the ML method and the Neighbor-Joining method, revealing similar clustering patterns.

**Figure 3:**
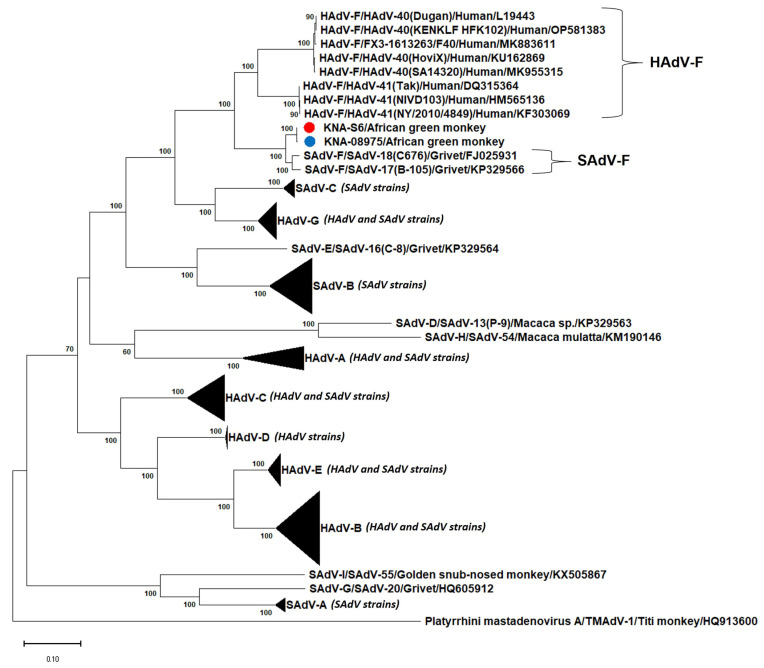
Phylogenetic analysis of the nearly complete deduced amino acid (aa) sequences of the putative DNA-dependent DNA polymerase (Pol) of simian adenovirus (SAdV) strains KNA-S6 and KNA-08975 (shown with red and blue circle, respectively) from African green monkeys (AGMs) with cognate sequences of human and simian mastadenoviruses (HAdV and SAdV, respectively). The name of the virus/host of detection are shown for the AGM SAdVs, whilst the *Mastadenovirus species*/name of the virus/host of detection/GenBank accession number have been mentioned for the HAdVs and other SAdVs. The clusters consisting of HAdV-A, -B, -C, -D, -E and -G and SAdV-A, -B, and -C strains were compressed. *Platyrrhini mastadenovirus A*/TMAdV-1/Titi monkey/HQ913600 was used as the outgroup sequence. Bootstrap values <60% are not shown. Scale bar, 0.1 substitutions per aa residue. The expanded phylogenetic tree is shown in Supplementary Figure S2.

Based on deduced aa identities, the nearly complete putative Pol sequences (1178 aa) of KNA-S6 and KNA-0875 were most closely related (99.75% identities) to each other (Table 2). With cognate Pol sequences from other AdVs, KNA-S6 and KNA-0875 shared maximum deduced aa identities of 96.11% and 96.03%, respectively, with SAdV-F/SAdV-18, followed by 96.02% and 95.94%, respectively, with SAdV-F/SAdV-17 (Table 2). Phylogenetically, the putative Pol of KNA-S6 and KNA-08975 grouped together, close to the SAdV-F cluster (SAdV-17 and -18), and near the HAdV-F cluster (Figure 3, Supplementary Figure S2). Although the Pol of KNA-S6 and KNA-08975 were most closely related to that of SAdV-F/SAdV-18, the cognate Pol sequence of SAdV-F/SAdV-18 was 9 aa longer than those of the AGM AdV strains (Supplementary Figure S3).

**Figure 4:**
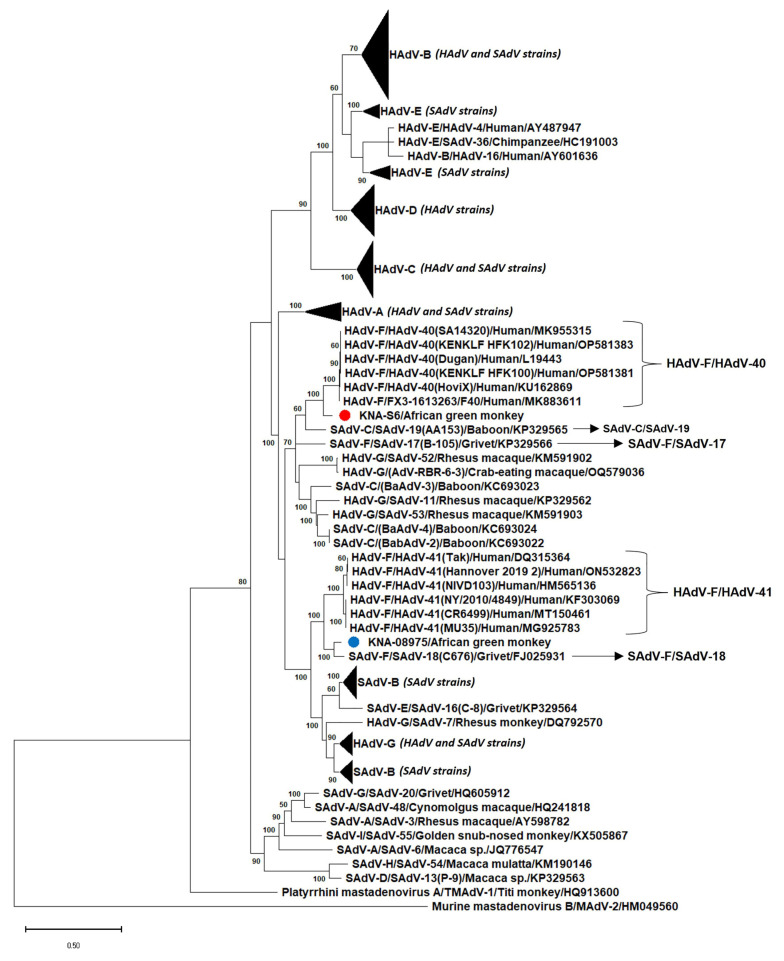
Phylogenetic analysis of the complete deduced amino acid (aa) sequences of the putative hexon of simian adenovirus (SAdV) strains KNA-S6 and KNA-08975 (shown with red and blue circle, respectively) from African green monkeys (AGMs) with those of human and simian mastadenoviruses (HAdV and SAdV, respectively). The name of the virus/host of detection are shown for the AGM SAdVs, whilst the *Mastadenovirus species*/name of the virus/host of detection/GenBank accession number have been mentioned for the HAdVs and other SAdVs. The clusters consisting of HAdV-A, -B, -C, -D, -E and -G and SAdV-B strains were compressed. *Murine mastadenovirus B*/MAdV-2/HM049560 was used as the outgroup sequence. Bootstrap values <60% are not shown. Scale bar, 0.5 substitutions per aa residue. The expanded phylogenetic tree is shown in Supplementary Figure S5.

The complete putative hexon of KNA-S6 and KNA-08975 was 925 aa and 923 aa in size, respectively, which was identical/comparable to those of HAdV-F/HAdV-41 (925 aa), and 8 and 6 aa, respectively, longer than those of SAdV-F/SAdV-17 and -18 (917 aa) (Supplementary Figure S4). The putative hexon of KNA-S6 and KNA-08975 shared deduced aa identities of 87.26% between themselves. Strain KNA-S6 shared deduced aa identities of 93% with the hexon sequences of HAdV-F/HAdV-40 strains, followed by identity of 90% with SAdV-C/SAdV-19 from a yellow baboon (*Papio cynocephalus*) [17], and <89% identities with other AdVs (Table 2). On the other hand, the putative hexon of KNA-08975 shared maximum aa identity of 94.79% with SAdV-F/SAdV-18, followed by ~90% identities with HAdV-F/HAdV-41 strains (Table 2). By phylogenetic analysis, the hexon aa sequences of some of the HAdVs and SAdVs including HAdV-F and SAdV-F strains did not exhibit species-specific clustering, corroborating previous observations (Figure 4) [16]. Phylogenetically, the putative hexon of KNA-S6 clustered with HAdV-F/HAdV-40 strains, near SAdV-C/SAdV-19, whilst KNA-08975 grouped with SAdV-F/SAdV-18 to form a single cluster, with HAdV-F/HAdV-41 as the nearest neighbor (Figure 4, Supplementary Figure S5). In the phylogenetic tree based on complete hexon coding (nt) sequences, the AGM AdVs retained similar clustering patterns, with KNA-S6 clustering near HAdV-F strains, whilst KNA-08975 clustered with SAdV-F/SAdV-18 (Supplementary Figure S6).

**Figure 5:**
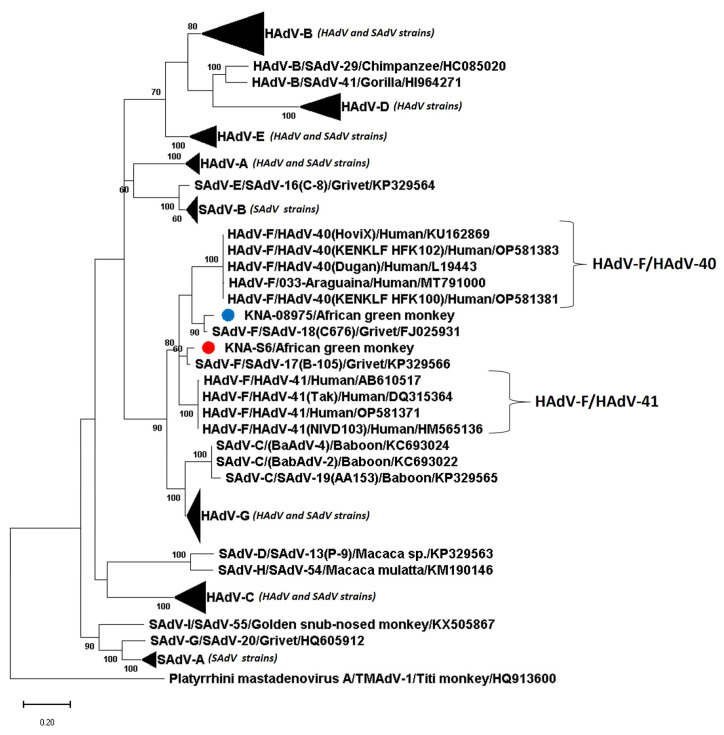
Phylogenetic analysis of the partial deduced amino acid (aa) sequences of the putative penton base of simian adenovirus (SAdV) strains KNA-S6 and KNA-08975 (shown with red and blue circle, respectively) from African green monkeys (AGMs) with cognate sequences of human and simian mastadenoviruses (HAdV and SAdV, respectively). The name of the virus/host of detection are shown for the AGM SAdVs, whilst the *Mastadenovirus species*/name of the virus/host of detection/GenBank accession number have been mentioned for the HAdVs and other SAdVs. The clusters consisting of HAdV-A, -B, -C, -D, -E and -G and SAdV-A, and -B strains were compressed. *Platyrrhini mastadenovirus A*/TMAdV-1/Titi monkey/HQ913600 was used as the outgroup sequence. Bootstrap values <60% are not shown. Scale bar, 0.2 substitutions per aa residue. The expanded phylogenetic tree is shown in Supplementary Figure S8.

The partial deduced aa sequences of the putative penton base (299 aa, corresponding to aa 210-aa 508 of the penton base sequence of SAdV-F/SAdV-18) of KNA-S6 and KNA-08975 retained the ‘RGD’ motif (binds to host cell integrins for triggering internalization [7]) that is present in SAdV-F and other primate AdVs, but absent in HAdV-F (Supplementary Figure S7) [7,46]. The partial penton base sequences of KNA-S6 and KNA-08975 shared 92.98% aa identities between themselves, and maximum identity of 97.66% and 97.32% with SAdV-F/SAdV-17 and SAdV-F/SAdV-18, respectively (Table 2). Before subjecting the partial penton base sequences of KNA-S6 and KNA-08975 to phylogenetic analysis, we confirmed that the clustering patterns observed with partial penton base sequences of published AdVs (used in the analysis) were similar to those based on complete sequences (Supplementary Figures S8 and S9). Phylogenetically, the partial penton base aa sequences of HAdV-F and SAdV-F strains formed a single clade, in which KNA-S6 grouped with SAdV-F/SAdV-17 to form a distinct branch, whilst KNA-08975 clustered with SAdV-F/SAdV-18, near HAdV-F/HAdV-40 (Figure 5, Supplementary Figure S8).

Based on analyses of deduced aa sequences of the three major AdV proteins (Pol, hexon and penton base), AGM AdV KNA-08975 was shown to be more closely related to SAdV-F/SAdV-18 (SAdV-F/SAdV-18-like) than those of other AdVs. On the other hand, the origin of KNA-S6 appear to be complex, with a SAdV-F/SAdV-18-like Pol, and a hexon and a penton base that was more closely related to HAdV-F/HAdV-40(HAdV-F/HAdV-40-like) and SAdV-F/SAdV-17, respectively. The AdV hexon has been proposed to be an active site for recombination events [3,14,15,16,17,18]. Although recombination analysis should be performed on complete genomes of AdVs [17], we attempted to evaluate the complete hexon coding sequence of KNA-S6 (and KNA-08975) for possible recombination events. However, inconclusive results were obtained with the RDP4 program.

The base composition (‘G + C’ %) of viral genomes has been considered as an additional parameter for classification of AdV species, and the ‘G + C’ content of partial AdV sequences have been shown to reflect those based on complete AdV genomes [1,17]. The ‘G + C’ content of SAdV-F (58.8% and 61.4% for SAdV-17 and -18, respectively) is higher than those of HAdV-F (~51%) [17]. The ‘G + C’ content of the putative Pol, hexon and penton base coding sequences of KNA-08975 (65%, 55%, and 60%, respectively) and the Pol and penton base coding sequences of KNA-S6 (65% and 59%, respectively) were comparable to those of cognate sequences of SAdV-F/SAdV-18 (66%, 57% and 63%, respectively), and higher than those of HAdV-F (54%, 49–54% and 50%, respectively). Interestingly, the ‘G + C’ composition of the KNA-S6 hexon (51%) was comparable to those of HAdV-F (49–50%), and lower than those of SAdV-F/SAdV-18 (57%). Based on sequence identities, phylogenetic analysis, and evaluation of ‘G + C’ content, it appears that recombination and cross-species transmission events involving HAdV-F- and SAdV-F-like viruses might have influenced the origin of AGM AdV KNA-S6.

## 4. Discussion

To our knowledge, this is the first report on detection and molecular characterization of AdVs from AGMs. Overall, high AdV detection rates (30.43%, 28/92 AGMs) were observed in the AGMs from St. Kitts. Based on analysis of the partial Pol- and hexon- aa sequences of a subset of AGM AdVs, and the nearly complete Pol, complete hexon and partial penton base sequences of two representative AdV strains (KNA-08975, group-I and KNA-S6, group-II), it appears that at least two AdV genetic variants (group-I: 7 AdVs with a SAdV-F/SAdV-18-like Pol and hexon, and group-II: 2 AdVs with a Pol and a hexon more closely related to that of SAdV-F/SAdV-18 and HAdV-F/HAdV-40, respectively, than other AdVs) might be circulating and even endemic (as both AdV variants were detected in samples from 2014–2015 and 2022) to the AGM population on St. Kitts. Several of the AdVs with a SAdV-F/SAdV-17/18-like partial Pol could not be amplified for the hexon gene, indicating that the levels of genetic diversity among AGM AdVs in St. Kitts might be higher than those reported in the present study.

The mastadenovirus species SAdV-F is understudied, represented by only two isolates (SAdV-17 and -18) that were detected in captive grivet monkeys during the 1950s [1,45,47,48,49], and since then, there has been no published reports on the identification of these viruses from NHPs (based on a PubMed search using keywords ‘simian/monkey/non-human primate/non-human primate/non-human primate adenovirus 17/18’, ‘adenovirus 17/18’, and/or ‘simian adenovirus F’). We reported SAdV-F-like AdVs for the first time in free-roaming NHPs and after ~ six decades from captive NHPs. The detection of SAdV-F-like AdVs and SAdV-F/SAdV-17/18 in related host species (AGMs and grivet monkeys, respectively, genus *Chlorocebus*) mirrored the hypothesis that AdVs have a narrow host range [1,2,3,4,14,15,17]. SAdV-F/SADV-17 and -18 were detected in the intestinal contents of grivet monkeys [47], whilst the SAdV-F-like AdVs from AGMs were identified in both fecal/rectal and nasal samples. There is a lack of information on the pathogenesis of SAdV-F/SAdV-17 and -18 [17,46], whilst the SAdV-F-like AdVs from this study were detected in apparently healthy AGMs, warranting studies on the tropism and disease-causing potential of SAdV-F in NHPs. The complete genomes of SAdV-17 and -18 were obtained from AdV isolates that have been propagated/maintained in cell cultures for years before they were sequenced [45,46]. On the other hand, the AdV genomes reported in this study were determined from wild-type AdVs detected in AGMs. Interestingly, the putative Pol and hexon sequences of SAdV-F/SAdV-18-like AdV KNA-08975 were 9 aa shorter and 6 aa longer, respectively, than those of SAdV-F/SAdV-18 (cell culture adapted strain C676 [45]).

Interestingly, analyses of the putative Pol, hexon and penton base sequences of AGM AdV group-II strain KNA-S6 (and partial Pol and hexon sequences of KNA-2-2022-rectal) indicated possible recombination and cross-species transmission events involving HAdV-F- and SAdV-F-like viruses. The group-I (KNA-08975) and -II (KNA-S6) representative samples were also screened with HAdV-F- and SAdV-F-specific hexon primers, respectively, ruling out mixed infections with viruses of the other group (Supplementary Table S1). KNA-S6 was detected in a free-roaming AGM trapped and sampled at the Southeastern peninsula of St. Kitts (Table 1). Although this area is not inhabited by humans, there are a few restaurants by the beach, and a harbor was being constructed at the time of trapping, which might have resulted in possible NHP-human contact. Furthermore, humans have been known to let loose their pet animals in this part of the island, and therefore, KNA-S6 could have been an abandoned pet monkey. On the other hand, KNA-2-2022-rectal was from a captive AGM at the BSF quarantine facility (Table 1). Since the AGM was sampled the next day after trapping, and was in quarantine during sampling, it is likely that the NHP acquired the virus outside the facility. The small Island of St. Kitts has a large population of AGMs (~40,000 [27,28,29]) that are in constant movement, often straying into human habitats, which might have facilitated cross-species transmission events of AdVs.

Taken together, our observations corroborated the hypothesis that the evolutionary pathways of HAdVs and SAdVs are intermingled, complicated by recombination and inter-species transmission events, especially between related AdV species, such as HAdV-F and SAdV-F [3,14,15,16,17,18,19,23,24,46]. The pathogenic potential of SAdV-F in NHPs (and humans) remains to be elucidated [17,45,46,47,48,49], whilst HAdV-F (HAdV-40 and -41) has been recognized as one of the leading global causes of viral diarrhea and deaths in young of humans, and HAdV-F-41 has been associated with severe pediatric hepatitis [51]. Therefore, (i) the present findings, (ii) several other observations that support zoonotic and anthroponotic transmission of SAdVs and HAdVs [3,9,14,15,16,17,22,23,24,25], and (iii) the threat of possible emergence of novel, virulent pathogens derived from recombination events involving HAdVs and SAdVs [3,14,15,22,52], underscore the importance of One-Health-based studies aimed at detection and molecular characterization of AdVs circulating in human and NHP populations, with implications on public health and animal health.

Although the present study provided first-time and important insights into AdVs circulating in captive and free-roaming AGMs, with evidence for possible recombination and interspecies transmission events involving HAdVs and SAdVs, there were limitations: (i) the study was based on a small sample size (92 AGMs); (ii) sampling lacked consistency (40 fecal samples from AGMs during 2014–2015, whilst nasal and rectal samples from 52 AGMs in 2022); (iii) although the AGM samples were screened using a *pol*-based nested PCR assay and a hexon-based PCR assay (both assays have been shown to be broad-spectrum for NHP AdVs [8,23,25,38]), revealing high detection rates (30.43%, *n* = 92 AGMs), these screening assays may not be as sensitive as qPCR assays; (iv) since samples KNA-S6 and KNA-08975 were not available in sufficient volumes, we could not determine the whole genomic sequences, or those of other important AdV genes (VA-RNA gene and the fiber gene) for both AdV strains. Of note, HAdV-F, SAdV-F/SAdV-17 and SAdV-F/SAdV-18 have been shown to possess 2, 3, and 1 fiber genes, respectively [46], and (v) we did not have access to samples from humans residing near the AGM trapping sites. Considering the detection of AGM AdVs with a SAdV-F-like Pol and HAdV-F-like hexon, it would have been interesting to investigate the local human population for AdVs.

## Figures and Tables

**Table 1:** Details of the African green monkeys (AGMs) that tested positive for adenoviruses in the Caribbean Island of St. Kitts.

AGM Number	Age, Gender	Adenovirus Positive Sample	Date of Sample Collection	Sampling/Trapping Location in St. Kitts
KNA-S2	Juvenile, male	Feces	12 July 2014	Southeast Peninsula ^1^
KNA-S6	Adult, male	Feces	15 November 2014	Southeast Peninsula ^1^
KNA-08972	Not available	Feces	9 September 2015	Behavioral Science Foundation ^2^
KNA-08973	Not available	Feces	2 October 2015	Behavioral Science Foundation ^2^
KNA-08975	Juvenile, male	Feces	19 October 2015	Behavioral Science Foundation ^2^
KNA-08977	Adult, male	Feces	19 October 2015	Behavioral Science Foundation ^2^
KNA-08980	Not available	Feces	9 October 2015	Behavioral Science Foundation ^2^
KNA-08982	Not available	Feces	6 October 2015	Behavioral Science Foundation ^2^
KNA-08984	Not available	Feces	9 October 2015	Behavioral Science Foundation ^2^
KNA-08985	Adult, female	Feces	15 October 2015	Behavioral Science Foundation ^2^
KNA-08994	Adult, female	Feces	15 October 2015	Behavioral Science Foundation ^2^
KNA-08995	Not available	Feces	27 October 2015	Behavioral Science Foundation ^2^
KNA-1-22	Adult, male	Nasal swab	24 September 2022	Monkey hill ^1^
KNA-2-22	Juvenile, male	Nasal and rectal swab	24 September 2022	Monkey hill ^1^
KNA-3-22	Adult, female	Rectal swab	24 September 2022	Monkey hill ^1^
KNA-5-22	Juvenile, male	Nasal swab	24 September 2022	Monkey hill ^1^
KNA-6-22	Adult, female	Rectal swab	24 September 2022	Monkey hill ^1^
KNA-7-22	Juvenile, male	Nasal and rectal swab	24 September 2022	Monkey hill ^1^
KNA-9-22	Adult, male	Nasal and rectal swab	24 September 2022	Monkey hill ^1^
KNA-11-22	Adult, female	Nasal swab	15 December 2022	Monkey hill ^1^
KNA-13-22	Juvenile, female	Rectal swab	15 December 2022	Monkey hill ^1^
KNA-14-22	Adult, male	Rectal swab	15 December 2022	Monkey hill ^1^
KNA-15-22	Juvenile, male	Nasal swab	15 December2022	Monkey hill ^1^
KNA-16-22	Adult, male	Nasal swab	15 December2022	Monkey hill ^1^
KNA-22-22	Adult, female	Rectal swab	19 December2022	Cedar Grove ^3^
KNA-25-22	Adult, female	Nasal swab	19 December2022	Cedar Grove ^3^
KNA-31-22	Adult, male	Rectal swab	20 December2022	Cedar Grove ^3^
KNA-38-22	Adult, male	Rectal swab	29 December2022	Green hill ^3^

^1^ The AGM was sampled at the trapping site. ^2^ The fecal samples were obtained from captive AGMs (housed in individual cages) at the quarantine facility of Behavioral Science Foundation (BSF), Estridge estate, St. Kitts. ^3^ The AGM was sampled immediately after arrival to the BSF quarantine facility from the trapping site ^3^.

**Table 2:** Deduced amino acid (aa) identities of the putative DNA-dependent DNA polymerase (Pol), hexon, and penton base proteins of simian adenovirus (SAdV) strains KNA-S6 and KNA-08975 from African green monkeys (AGMs) with those of *Human mastadenovirus-F* (HAdV-F) and *Simian mastadenovirus-F* (SAdV-F) strains. The *Mastadenovirus species*/name of the virus/host of detection/GenBank accession number are shown for the HAdV-F and SAdV-F strains.

Putative Adenovirus Protein	Deduced aa Identities (%)	
	KNA-S6	KNA-08975
DNA-dependent DNA polymerase (Pol) ^1^	➢ 99.75% with KNA-08975 ➢ 96.11% with SAdV-F/SAdV-18(C676)/Grivet/FJ025931 ➢ 96.02% with SAdV-F/SAdV-17(B-105)/Grivet/KP329566 ➢ <86% with other AdV strains 85.50% with HAdV-F/HAdV-41(Tak)/Human/DQ315364 84.31% with HAdV-F/HAdV-40(Dugan)/Human/L19443	➢ 96.03% with SAdV-F/SAdV-18(C676)/Grivet/FJ025931 ➢ 95.94% with SAdV-F/SAdV-17(B-105)/Grivet/KP329566 ➢ <86% with other AdV strains 85.33% with HAdV-F/HAdV-41(Tak)/Human/DQ315364 84.22% with HAdV-F/HAdV-40(Dugan)/Human/L19443
Hexon ^2^	➢ 87.26% with KNA-08975 ➢ 93.20% with HAdV-F/FX3-1613263/F40/Human/MK883611 ➢ 93.09% with HAdV-F/HAdV-40(Dugan)/Human/L19443 ➢ 90.26% with SAdV-C/SAdV-19(AA153)/Baboon/KP329565 ➢ 88.72% with SAdV-F/SAdV-18(C676)/Grivet/FJ025931 ➢ 88.25% with SAdV-F/SAdV-17(B-105)/Grivet/KP329566 ➢ 87.33% with HAdV-F/HAdV-41(Tak)/Human/DQ315364	➢ 94.79% with SAdV-F/SAdV-18(C676)/Grivet/FJ025931 ➢ 90.35% with HAdV-F/HAdV-41(CR6499)/Human/MT150461 ➢ 90.35% with HAdV-F/HAdV-41(MU35)/Human/MG925783 ➢ 90.03% with HAdV-F/HAdV-41(Tak)/Human/DQ315364 ➢ 86.81% with SAdV-F/SAdV-17(B-105)/Grivet/KP329566 ➢ 85.85% with HAdV-F/HAdV-40(Dugan)/Human/L19443
Penton ^3^	➢ 92.98% with KNA-08975 ➢ 97.66% with SAdV-F/SAdV-17(B-105)/Grivet/KP329566 ➢ 93.65% with HAdV-F/HAdV-41(Tak)/Human/DQ315364 and several other HAdV-F/HAdV-41 strains ➢ 92.98% with SAdV-F/SAdV-18(C676)/Grivet/FJ025931 ➢ 91.30% with HAdV-F/HAdV-40(Dugan)/Human/L19443	➢ 97.32% with SAdV-F/SAdV-18(C676)/Grivet/FJ025931 ➢ 93.98% with SAdV-F/SAdV-17(B-105)/Grivet/KP329566 ➢ 91.30% with HAdV-F/033-Araguaina/Human/MT791000 ➢ 90.97% with HAdV-F/HAdV-40(Dugan)/Human/L19443 ➢ 90.97% with HAdV-F/HAdV-41(Tak)/Human/DQ315364

^1^ Based on nearly complete deduced aa sequences (corresponding to aa 7- aa 1189 of strain SAdV-F/SAdV-18(C676)/Grivet/FJ025931) of the putative Pol of AGM adenovirus (AdV) strains KNA-S6 (1178 aa in size) and KNA-08975 (1178 aa) with cognate sequences of other AdV strains. ^2^ Based on complete deduced aa sequences of the putative hexon of AGM adenovirus strains KNA-S6 (925 aa in size) and KNA-08975 (922 aa) with those of other AdV strains. ^3^ Based on partial deduced aa sequences (corresponding to aa 210- aa 508 of strain SAdV-F/SAdV-18(C676)/Grivet/FJ025931) of the putative penton base of AGM adenovirus strains KNA-S6 (299 aa in size) and KNA-08975 (299 aa) with cognate sequences of other AdV strains.

## Data Availability

The data presented in this study are available in this article and Supplementary Table S1 and Figures S1–S9.

## Supplementary Materials

The following supporting information can be downloaded at: https://www.mdpi.com/article/10.3390/v15071605/s1, Supplementary Table S1: Primers used to obtain the complete/nearly complete DNA-dependent DNA polymerase and hexon coding sequences, and partial penton base coding sequences of simian adenovirus (SAdV) strains KNA-S6 and KNA-08975 detected in African green monkeys from St. Kitts; Supplementary Figure S1: Flow chart summarizing the work pipeline and PCR results from the present study; Supplementary Figure S2: Expanded version of Figure 3; Supplementary Figure S3: Multiple alignment of the nearly complete deduced amino acid (aa) sequences of the putative DNA-dependent DNA polymerase (Pol) of simian adenovirus (SAdV) strains KNA-S6 and KNA-08975 with cognate sequences of SADV-F/SAdV-17 strain B-105 (GenBank accession number KP329566), SAdV-F/SAdV-18 strain C676 (FJ025931), Human AdV-F (HAdV-F)/HAdV-40 isolate Dugan (L19443), and HAdV-F/HAdV-41 isolate Tak (DQ315364); Supplementary Figure S4: Multiple alignment of the complete deduced amino acid (aa) sequences of the putative hexons of simian adenovirus (SAdV) strains KNA-S6 and KNA-08975 with those of SADV-F/SAdV-17 strain B-105 (GenBank accession number KP329566), SAdV-F/SAdV-18 strain C676 (FJ025931), Human AdV-F (HAdV-F)/HAdV-40 isolate Dugan (L19443), and HAdV-F/HAdV-41 isolate Tak (DQ315364); Supplementary Figure S5: Expanded version of Figure 4; Supplementary Figure S6: Phylogenetic analysis of the complete hexon coding sequences of simian adenovirus (SAdV) strains KNA-S6 and KNA-08975 (shown with red and blue circles, respectively) with those of human adenoviruses (HAdVs) and other SAdVs; Supplementary Figure S7: Multiple alignment of the partial deduced amino acid (aa) sequences of the putative penton bases of simian adenovirus (SAdV) strains KNA-S6 and KNA-08975 with cognate sequences of SADV-F/SAdV-17 strain B-105 (GenBank accession number KP329566), SAdV-F/SAdV-18 strain C676 (FJ025931), Human AdV-F (HAdV-F)/HAdV-40 isolate Dugan (L19443), and HAdV-F/HAdV-41 isolate Tak (DQ315364); Supplementary Figure S8: Expanded version of Figure 5; Supplementary Figure S9: Phylogenetic analysis of the complete deduced amino acid (aa) sequences of the putative penton bases of simian adenoviruses (SAdVs) and human adenoviruses (HAdVs).
