# Supplementary material for: Detection and Molecular Characterization of Adenoviruses in Captive and Free-Roaming African Green Monkeys (Chlorocebus sabaeus): Evidence for Possible Recombination and Cross-Species Transmission
Source: Viruses. 2023 Jul 22;15(7):1605. doi: 10.3390/v15071605 (PMC10385324; doi:10.3390/v15071605)
Supplement: Supplementary file 1 [file viruses-15-01605-s001.zip › viruses-2478472-supplementary.pdf]

Table S1. Primers used to obtain the complete/nearly complete DNA-dependent DNA polymerase and hexon coding sequences, and partial penton base coding sequences of simian adenovirus (SAdV) strains KNA-S6 and KNA-08975 detected in African green monkeys from St. Kitts. Primers employed in semi-nested PCR assays to amplify a region of the putative hexon that is genetically divergent between SAdV-17, -18 and *Human mastadenovirus-F* strains (HAdV-F) are highlighted with yellow. Forward primers (used in combination with 2 reverse primers designed from partial penton base sequences of KNA-S6 and KNA-08975) that failed to amplify the 5′- region of the penton base are shown with red font.

Target gene	Primer name	Primer sequence [1] (5′-3′)	Primer position [2]
	NHP-pol-5ENDF	TCTACGGCATCTCGATCCAGCAG	nt 5228-nt 5250
	NHP-pol-5ENDR	AAGCTCTACGCCCTCAAGTGCCTC	nt 5591-nt 5569
	NHP-pol-5261F	TGCGGGGGTTGGGRCGGCTTT	nt 5261-nt 5281
	NHP-pol-5283F	GCTGTASGGBACCAGBCGGTG	nt 5283-nt 5303
	NHP-pol-5946R	GGTGGATCCCCTGGTGGAGAACG	nt 5946-nt 5924
	NHP-pol-5800F	GATTTGAGCGCGCGCTGCTCCAG	nt 5800-nt 5822
	NHP-pol-6954R	CTCCAACTCSCACGCCATCTT	nt 6954-nt 6934
	NHP-pol-6976R	TCTTYCAGCGSCCCACCATCTC	nt 6976-nt 6955
	NHP-pol-6721F	CCCCAGGGCATGGGRTGGGT	nt 6721-nt 6740
	NHP-pol-6742F	AGGGCCGAGGCGTACATGCC	nt 6742-nt 6761
**DNA-dependent DNA polymerase**	NHP-pol-7983R	CACCTACGACGTVGARACCTACAC	nt 7983-nt 7960
	NHP-pol-7751F	TCTGGAGGCGGTCGCGGTAGT	nt 7751-nt 7771
	NHP-pol-7774F	CGGAACTTTTGCCCACCGCCA	nt 7774-nt 7795
	NHP-pol-8817R	CTGTYGAACTCTCTTTCAGGTTC	nt 8817-nt 8795
	NHP-pol-8752R	CAGGACCTCAACCGCCGCGT	nt 8752-nt 8733
	NHP-hex-18098F	AACAGCATYGTGGGYCTGGG	nt 18098-nt 18117
	NHP-hex-18582R	CGGTCCAGCACGCCGCGGATGTC	nt 18582-nt 18560
	NHP-hex-18625R	GGAGTTGTAAGCGGTGCCGGAGTA	nt 18625-nt 18602
	NHP-hex-18253F	CAAGATGGCCACCCCCTCSATGATGC	nt 18253-nt 18278
	NHP-hex-18308F	CAGGACGCCTCGGAGTACCTGAGC	nt 18308-nt 18331
	NHP-hex-18837R [3]	TCTCCGGCTTCGCTGTTCCACTG [3]	nt 18837-nt 18815
	NHP-hex-18836R [4]	GCGTGGGATCAATATTCCATTGCG [4]	nt 18836-nt 18813
	NHP-hex-18494F	TACAAAGTGCGCTTCACGCTGG	nt 18494-nt 18515
	NHP-hex-18524F	GACAACCGCGTGCTGGACATG	nt 18524-nt 18544
	NHP-hex-19337R	GGTCATAGCTGTCYACGGCCTG	nt 19337-nt 19316
	NHP-hex-19100F [3]	CAGCAGGCGGCTCCCAACAGAC [3]	nt 19100-nt 19117
	NHP-hex-19177F [3]	TGGAAATATGGGAGTTCTTGCGGG [3]	nt 19177-nt 19200
	NHP-hex-19174F [4]	CACTGGTAATATGGGGGTCTTGGC [4]	nt 19174-nt 19197
	NHP-hex-19192F [4]	CTTGGCAGGTCAGGCTTCGCAAC [4]	nt 19192-nt 19213
	NHP-hex-20006R	AGTTGGCGTAVAGGTTRATGCTGTC	nt 20006-nt 19982
**Hexon**	NHP-hex-19883F	TCCTACACCTACGAGTGGAACTT	nt 19883-nt 19905
	NHP-hex-20887R	CTCATCCATGGGGTCCACCTC	nt 20887-nt 20867
	NHP-hex-20946R	TGCGGCTGGTGGATGCGCAC	nt 20946-nt 20927
	NHP-hex-20783F	AACTTCATGTCCATGGGGGC	nt 20783-nt 20802
	NHP-hex-21114R	AAGCGYTTGTCRAASGTGCCCA	nt 21114-nt 21093
	NHP-hex-21162R	GTGTTRACRATGGCGCASGCCA	nt 21162-nt 21141
	NHP-pen-13461	TGGARAGCCTRGTRGAYAAGATGAA	nt 13461- nt 13485
	NHP-pen-13496	ACSTACGCSCAGGAGCAGCGGG	nt 13496- nt 13517
	NHP-pen-13610	AGCGTGTTGGACTTGGGGGG	nt 13610-nt 13629
	NHP-pen-13658	GCYCAYCTGCGSCCKCAGGG	nt 13658-nt 13677
	NHP-pen-14407F	GAAGTTYGACACGCGCAACTTCCG	nt 14407-nt 14430
	NHP-pen-14462F	ATGCCSGGCGTGTACACCAACGAG	nt 14462-nt 14485
	NHP-pen-15243R	GCGTCRGTGATGGTCACGCGC	nt 15243-nt 15223
**Penton base**	NHP-pen-15210R	ATRCTGCTGCGCAGCGGCAGGG	nt 15210-nt 15189
	NHP-pen-15036F	ACGAGCAGGCCGTCTACTCGCAGC	nt 15036-nt 15059
	NHP-pen-15446R	CGGTAGTGGCCGCGGACGCG	nt 15446-nt 15427

NHP-pen-15404R	CGCTTGGCGCCGCCGTACAT	nt 15404-nt 15385

[1] The primers were designed following multiple alignment of the complete genomic sequences of SAdV-18 strain C676 (GenBank accession number FJ025931), SAdV-17 strain B-105 (KP329566), and HAdV-F strains (HAdV-40 isolates Dugan (L19443) and SA12680 (MK883611), and HAdV-41 isolate Tak (DQ315364)).
[2] Nucleotide positions are those of SAdV-18 (FJ025931).
[3, 4] Primers specific to SAdV strain KNA-08975 [3] and KNA-S6 [4] (designed from partial hexon sequences of respective virus strains), respectively.

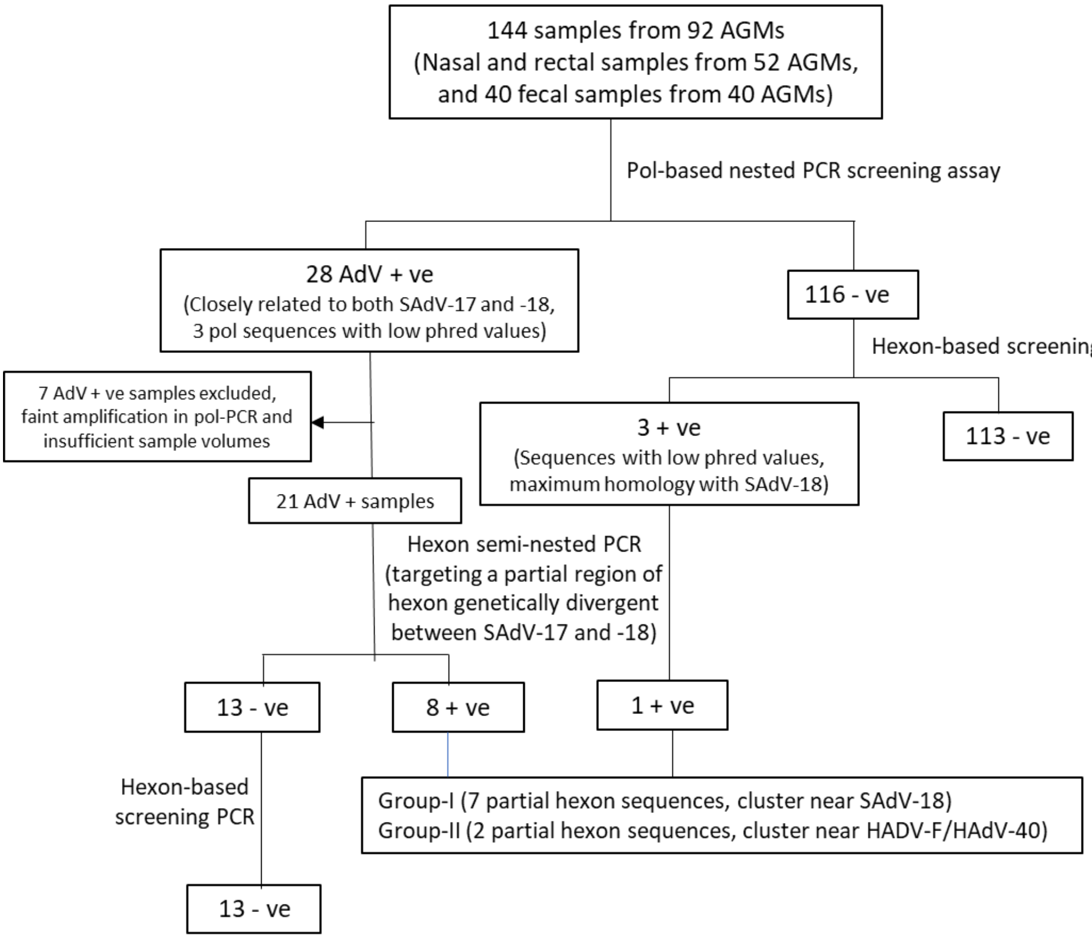

Figure S1. Flow chart summarizing the work pipeline and PCR results from the present study.

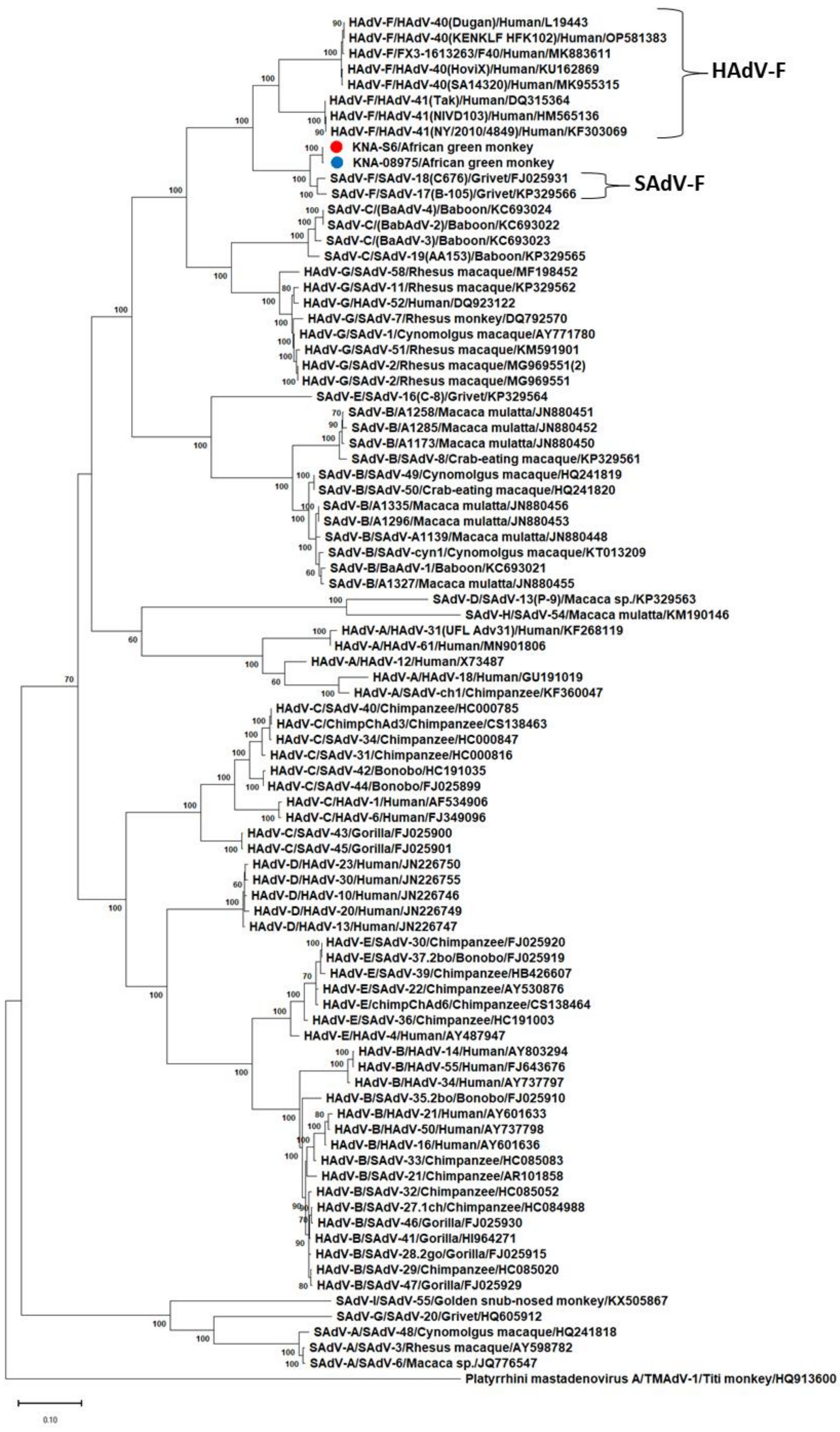

Figure S2. Expanded version of figure 3.

```
KNA-S6              ------PGAGGLHAAEAHPGTQPPRRRARQSAASPAPAAAGAPRRRAVAAAAGGTRTPAA
54
KNA-08975           ------PGAGGLHAAEAHPGTQPPRRRARQSAASPAPAAAGAPRRRAVAAAAGGTRTPAA
54
SAdV-F/SAdV-18      MALVPSPGTGGLHAAEAHPGPQPPRRRARQSAASSAPAAAGAPRRRAAAAHAGGTRTPAA
60
SADV-F/SAdV-17      MALVPSPGTGGLHAEKAHPGPQPPRRRARQSAASPAPAAAGAPRRRAAGAHAGGSRTPAA
60
HAdV-F/HAdV-40      MALVPSPRAGGFLPAETHSGPQPPRRRVRQSTAGAAPTATRAPRRRAATASPGEPPSTTA
60
HAdV-F/HAdV-41      MALVPSPGTLGLHSTKTYPGTQPPRCRARQSAAGSASTATRAPGQRASTSSSGGSRTATA
60
                          * : *: . :::.*.**** *.***:*..*.:*: ** :**  : .* . :.:*

KNA-S6              ARRPTAANHVS-----PAYRRHRGTIVAGRGHALLYAVDTSTNEPLEFKYHQRLAPALTR
109
KNA-08975           ARRPTAANHVS-----PAYRRHRGTIVAGRGHALLYAVDTSTNEPLEFKYHQRLAPALTR
109
SAdV-F/SAdV-18      ARRQAQAGDVTSPPEYATYRRHRGTIVAARGQALLYAIDTSTNEPLEIKYHQRLAPALTR
120
SADV-F/SAdV-17      ARRQAAADVN--PPEHATYRRHRGTIVAARGHGLLYAIDTSTNEPLEIKYHQRLAPALTR
118
HAdV-F/HAdV-40      SGRPPAANNVS----LTPNSRLRGTIVAPRGQGLLYAIDTATNSPMEIKFHRRLASALTR
116
HAdV-F/HAdV-41      PRRPSASNDVN----PPSTFRLRGTIVASRGQGLLYAIDTSTNSPLEIKFHQRLASALTR
116
                    . * . :.        ..  * ****** **:.****:**:**.*:*:*:*:***.****

KNA-S6              LLQVHRRTLPVDLNEAFLNSLDAAQIRALALRLRPPRVDIWTCGSRGVVTPSVLHPQQER
169
KNA-08975           LLQVHRRTLPVDLNEAFLNSLDAAQIRALALRLRPPRVDIWTCGSRGVVTPSVLHPQQER
169
SAdV-F/SAdV-18      LLQVHRRTLPVDLSEAFLDSLDAAQIRTLALSLRPPRVDIWTCGSRGVVTPSVLHPQQER
180
SADV-F/SAdV-17      LLQVHRRTLPVDLDEAFLDSLDAAHIRTLALRLRPPRVDIWTCGLRGIVTPSVLHPQQER
178
HAdV-F/HAdV-40      LLQVNLRSVPADLNEAFLDSLDSSQIRTLALKLKVPRVEVWTCGSRGVVVPSIIHPQQER
176
HAdV-F/HAdV-41      LLQVNLRSLPAGLSEAFLDSLDSSQIRSLALRLQPPRVEVWTCASRGIVTPSVILPQQER
176
                    ****: *::*..*.****:***:::**:*** *: ***::***. **:*.**:: *****

KNA-S6              AGAEEHDEAEGQRAEAPLDCPLRFLVRGRRVHLVQEVQSVQRCEHCARFYKYQHECTVRR
229
KNA-08975           AGAEEHDEAEGQRAEAPLDCPLRFLVRGRRVHLVQEVQSVQRCEHCARFYKYQHECTVRR
229
SAdV-F/SAdV-18      AGAEEHDEAEGQRAEAPLDCPLRLLVRGRRVHLVQEVQSVQRCEYCARFYKYQHECTVRR
240
SADV-F/SAdV-17      AGAEEHDEAEGQHAEAPLNCPLRLLVRGRRVHLVQEVQSVQRCEYCARFYKHQHECTVRR
238
HAdV-F/HAdV-40      AGAEEGDEGERQDTEDFLNFPLRFLVRGRQVHLIQEMQSVQRCEYCARFYKYQHECTVRR
236
HAdV-F/HAdV-41      AGAEENNEGERQSAQEPLNFPLRFLVRGRQVHLIQEVQNVQRCEYCARFYKYQHECTVRR
236
                    ***** :*.* * ::  *: ***:*****:***:**:*.*****:******:********

KNA-S6              RDFYFHHVNAHSSGWWQEINFFPLGSHPRTERLFVTYDVETYTWMGAFGKQLVPFMLVMH
289
KNA-08975           RDFYFHHVNAHSSGWWQEINFFPLGSHPRTERLFVTYDVETYTWMGAFGKQLVPFMLVMH
289
SAdV-F/SAdV-18      RDFYFHHVNAHSSGWWQEINFFPLGSHPRTERLFVTYDVETYTWMGAFGKQLVPFMLVMH
300
SADV-F/SAdV-17      RDFYFHHVNAHSSGWWQQINFFPLGSHPRTERLFVTYDVETYTWMGAFGKQLVPFMLVMH
298
HAdV-F/HAdV-40      RDFYFHHINAHSSGWWQKINFFPIGSHPRVERLFVTYDVETYTWMGAFGKQLVPFMLVMH
296
HAdV-F/HAdV-41      RNFYFHHINAQSSGWWQEINFFPIGSHPRVERLFVTYDVETYTWMGAFGKQLVPFMLVMH
296
                    *:*****:**:******:*****:*****.******************************
```

```
KNA-S6              LSGEEALVEEACRLAREQSWDAWGEDGRTFYVVTPEKMAVGKKFRDYRDRLQTALALQLW
349
KNA-08975           LSGEEALVEEACRLAREQSWDAWGEDGRTFYVVTPEKMAVGKKFRDYRDRLQTALALQLW
349
SAdV-F/SAdV-18      LSGEEALVEEACRLAREQNWDAWGQDGRTFYVVTPEKMAVGKKFRDYRDRLQTQLALQLW
360
SADV-F/SAdV-17      LSGEETLVEEACRLAREQSWDAWGEDGRTFYVVTPEKMAVGKKFRDYRDRLQTALALQLW
358
HAdV-F/HAdV-40      LSGEEALVKEACRLACELQWDTWGNDERTFYVVTPEKLAVGKKFREYRNRLQAHFALQLW
356
HAdV-F/HAdV-41      LSGDDTLVEEACRLAHELQWDAWGNDGHTFYVVTPEKMAVGKKFRNYRDRLQTHFATQLW
356
                    ***:::**:****** * .**:**:* :*********:*******:**:***: :* ***

KNA-S6              RGFLQANPQVADWARQELGLYSPDCLTYEELKKAPKLQGRPRFVELYIVGHNINGFDEIV
409
KNA-08975           RGFLQANPQVADWARQELGLYSPDCLTYEELKKAPKLQGRPRFVELYIVGHNINGFDEIV
409
SAdV-F/SAdV-18      RGFLQANPQVAEWARQELGLYSPDCLTYEELKKAPKLQGRPRFVELYIVGHNINGFDEIV
420
SADV-F/SAdV-17      RGFLQANPQVAEWARQELGLYSPDCLTYEELKKAPKLQGRPRFVELYIVGHNINGFDEIV
418
HAdV-F/HAdV-40      RGFLAANPQLAEWACLEMGLFSPDYLTYEELQKAPKLQGRPRFLELYIVGHNINGFDEIV
416
HAdV-F/HAdV-41      QAFLAANPQVAEWARLEMGLFSPDNLTYEELKKAPKLQGTPRFVELYIVGHNINGFDEIV
416
                    :.** ****:*:**  *:**:*** ******:******* ***:****************


KNA-S6              LAAQVINNRSDVPGPFRITRNFMPRAGKILFNDVTFALPNPRSQKRTDYGLWERGACDDT
469
KNA-08975           LAAQVINNRSDVPGPFRITRNFMPRAGKILFNDVTFALPNPRSQKRTDYGLWERGACDDT
469
SAdV-F/SAdV-18      LAAQVINNRSDVPGPFRITRNFMPRAGKILFNDVTFALPNPRSQKRTDYGLWERGACDDT
480
SADV-F/SAdV-17      LAAQVINNRSDVPGPFRITRNFMPRAGKILFNDVTFALPNPRSQKRTDYGLWERGACDDT
478
HAdV-F/HAdV-40      LAAQVINNRSDVPGPFKITRNFMPRAGKILFNDITFALPNPSSKKRTDYRLWEQGACDDS
476
HAdV-F/HAdV-41      LAAQVINNRSDVPGPFKITRNFMPRAGKILFNDITFALPNPLSKKRTDYHLWEQGACDDT
476
                    ****************:****************:******* *:***** ***:*****:

KNA-S6              DFRFQFLKVMVRDTFALTHTSLRKAAQAYALPVEKGCCPYRAVNRFYMLGSYLADERGFP
529
KNA-08975           DFRFQFLKVMVRDTFALTHTSLRKAAQAYALPVEKGCCPYRAVNRFYMLGSYLADERGFP
529
SAdV-F/SAdV-18      DFRFQFLKVMVRDTFALTHTSLRKAAQAYSLPVEKGCCPYRAVNRFYMLGSYLADERGFP
540
SADV-F/SAdV-17      DFRFQFLKVMVRDTFALTHTSLRKAAQAYSLPVEKGCCPYRAVNRFYMLGSYLADERGFP
538
HAdV-F/HAdV-40      DFKYQFLKVMVRDTFALTHTSLRKAAQAYTLPVEKGCCPYKAVNEFYMLGSYRADERGFP
536
HAdV-F/HAdV-41      DFKHQFLKVMVRDTFALTHTSLRKAAQAYALPVEKGCCPYKAVNQFYMLGSYRADERGFP
536
                    **:.*************************:**********:***.******* *******

KNA-S6              AEEYWKDREEYLLNRELWEKKQEPRYDLVRETLDYCALDVLVTAALVQKLRESYAQFIHD
589
KNA-08975           AEEYWKDREEYLLNRELWEKKQEPRYDLVRETLDYCALDVLVTAALVQKLRESYAQFIHD
589
SAdV-F/SAdV-18      AEEYWKDREEYLLNRELWEKKQEPRYDLVRETLDYCALDVLVTAALVQKLRDSYAQFVHD
600
SADV-F/SAdV-17      AEEYWKDREEYLLNRELWEKKQEPRYDLVRETLDYCALDVLVTAALVQKLRDSYAQFVHD
598
HAdV-F/HAdV-40      AEDYWKDREEYLLNRELWEKKQCPHYDLVRETLDYCALDVLVTAALVQKLRESYAQFIRD
596
HAdV-F/HAdV-41      AEDYWKDREEYLLNRELWEKKQQSRYDLIRETLDYCALDVLVTAALVQKLRESYAQFICD
596
                    **:******************* .:***:**********************:*****: *
```

```
KNA-S6              AVGLPEASFNIFQRPTISSNSHAIFRQVTYRAVRPQRADLGGGLLAPSHEMYDYVRASIR
649
KNA-08975           AVGLPEASFNIFQRPTISSNSHAIFRQVTYRAVRPQRADLGGGLLAPSHEMYDYVRASIR
649
SAdV-F/SAdV-18      AVGLPEASFNIFQRPTISSNSHAIFRQVTYRAVRPQRGDLGGGLLAPSHEMYDYVRASIR
660
SADV-F/SAdV-17      AVGLPEASFNIFQRPTISSNSHAIFRQVTYRAVKPQRGDLGGGLLAPSHEMYDYVRASIR
658
HAdV-F/HAdV-40      AVGLPEASFNVFQRPTISSNSHAIFRQILYRTVKPQRSDLGGSLLAPSHEMYDYVRASIR
656
HAdV-F/HAdV-41      AVGLPEAKFNVFQRPTISSNSHAIFRQILYRSVKPKRSDLGNGLLAPSHEMYDYVRASIR
656
                    *******.**:****************: **:*:*:*.***..*****************

KNA-S6              GGRCYPTYLGVLREPLYVYDICGMYASALTHPMPWGAPLNPYERALAVRDWERALADPAV
709
KNA-08975           GGRCYPTYLGVLREPLYVYDICGMYASALTHPMPWGAPLNPYERALAVRDWERALADPAV
709
SAdV-F/SAdV-18      GGRCYPTYLGVLREPLYVYDICGMYASALTHPMPWGAPLNPYERALAVRDWERALADPAV
720
SADV-F/SAdV-17      GGRCYPTYLGVLREPLYVYDICGMYASALTHPMPWGAPLNPYERALAVRDWERALADPAV
718
HAdV-F/HAdV-40      GGRCYPTYIGVLREPLYVYDICGMYASALTHPMPWGFPLNPYERALAVRDWEHALLQVGT
716
HAdV-F/HAdV-41      GGRCYPTYIGVLREPLYVYDICGMYASALTHPMPWGSPLNPYERALAVRDWERALLQVDT
716
                    ********:*************************** ***************:** :  .

KNA-S6              SIDYFDRQLLPGIFTIDADPPAEDQLDVLPPFCSRKGGRLCWTNEPLRGEVATSVDLITL
769
KNA-08975           SIDYFDRQLLPGIFTIDADPPAEDQLDVLPPFCSRKGGRLCWTNEPLRGEVATSVDLITL
769
SAdV-F/SAdV-18      AIDYFDRHLLPGIFTIDADPPAEDQLDVLPPFCSRKGGRLCWTNEPLRGEVATSVDLITL
780
SADV-F/SAdV-17      AIDYFDRHLLPGIFTIDADPPAEDQLDVLPPFCSRKGGRLCWTNEPLRGEVATSVDLITL
778
HAdV-F/HAdV-40      PIDYFNRTLLPGIFTIDADPPPENLLDVLPPLCSRKGGRLCWTNEPLRGEVVTSVDLITL
776
HAdV-F/HAdV-41      PIDYFNHVLLPGIFTIDADPPSENLLDVLPPYCSRKGGRLCWTNEPLRGEVATSIDLITL
776
                    .****:: *************.*: ****** *******************.**:*****

KNA-S6              HNRGWRVRLLPDERATVFPEWRCVAREYVQLNIAAKERADREKNQTLRSIAKLLSNALYG
829
KNA-08975           HNRGWRVRLLPDERATVFPEWRCVAREYVQLNIAAKERADREKNQTLRSIAKLLSNALYG
829
SAdV-F/SAdV-18      HNRGWRVRLLPDERATVFPEWRCVAREYVQLNIAAKERADREKNQTLRSIAKLLSNALYG
840
SADV-F/SAdV-17      HNRGWRVRLLPDERATVFPEWRCVAREYVQLNIAAKERADREKNQTLRSIAKLLSNALYG
838
HAdV-F/HAdV-40      HNRGWHVRLLPDERATVFPEWRCVAKEYVHLNITAKERADREKNQTLRSIAKLLSNALYG
836
HAdV-F/HAdV-41      HNRGWQVRLLPDERTTVFPEWRCVARKYVQLNIAAKERADREKNQTLRSIAKLLSNALYG
836
                    *****:********:**********::**:***:**************************


KNA-S6              SFATKLDNKKIVFSDQMDPATVKSIAAGQVNIKSTSFVETDTLSAEVMPAFQRAYSPEQL
889
KNA-08975           SFATKLDNKKIVFSDQMDPATVKSIAAGQVNIKSTSFVETDTLSAEVMPAFQRAYSPEQL
889
SAdV-F/SAdV-18      SFATKLDNKKIVFSDQMDPATVKSIAAGQVNIKSTSFVETDTLSAEVMPAFQRAYSPEQL
900
```

```
SADV-F/SAdV-17      SFATKLDNKKIVFSDQMDPATVKSIAAGQVNIKSTSFVETDTLSAEVMPAFQRAYSPEQL
898
HAdV-F/HAdV-40      SFATKLDNKKIVFSDQMDSATIKSIAAGQINIKSTSFVETDTLSAEVMPTFQRAYSPEQL
896
HAdV-F/HAdV-41      SFATKLDNKKIVFSDQMDPATIKSIAAGQIKIKSTSFVETDTLSAEVMPAFQRAYSPEQL
896
                    ******************.**:*******::******************:**********

KNA-S6              DLVHSDAEESDGETGHAPFYKPTRDPDGHVTYTYKPITFMDAEEDDLCLHTLEKVDPLVE
949
KNA-08975           DLVHSDAEESDGETGHAPFYKPTRDPDGHVTYTYKPITFMDAEEDDLCLHTLEKVDPLVE
949
SAdV-F/SAdV-18      DLVHSDAEESDGEAGHAPFYKPTRDPDGHVTYTYKPITFMDAEEDDLCLHTLEKVDPLVE
960
SADV-F/SAdV-17      DLVHSDAEESDGEAGHAPFYKPTRDPDGHVTYTYKPITFMDAEEDDLCLHTLEKVDPLVE
958
HAdV-F/HAdV-40      AVVHSDAEESDEEPGHAPFYTPTHKPNDHVTYTYKPITFMDAEEDDLCLHTLEKVDPLVE
956
HAdV-F/HAdV-41      ALAHSDAEESDEERGHA-LYTPTQDPKGHVTYTYKPITFMDAEEDDLCLHTLEKVDPLVE
955
                     :.******** * *** :*.**:.*..********************************

KNA-S6              NDRYPSQIASFVLAWTRAFVSEWSEFLHAEDRGIPLEQRALKSVYGDTDSLFVTEAGRRL
1009
KNA-08975           NDRYPSQIASFVLAWTRAFVSEWSEFLHAEDRGIPLEQRALKSVYGDTDSLFVTEAGRRL
1009
SAdV-F/SAdV-18      NDRYPSQIASFVLAWTRAFVSEWSEFLHAEDRGIPLEQRALKSVYGDTDSLFVTEAGRRL
1020
SADV-F/SAdV-17      NDRYPSQIASFVLAWTRAFVSEWSEFLHAEDRGIPLEQRALKSVYGDTDSLFVTEAGRRL
1018
HAdV-F/HAdV-40      NNRYPSQIASFVLAWTRAFVSEWSEILYAEDRGTPLEQRTLKSVYGDTDSLFVTEAGYRL
1016
HAdV-F/HAdV-41      NDRYPSQIASFVLAWTRAFVSEWSEFLYAEDRGTPLEQRTLKSVYGDTDSLFVTEAGHRL
1015
                    *:***********************:*:***** *****:***************** **

KNA-S6              METRGKKRIKKHGGQLVFDPQRPELTWLVECETTCAQCGADAFSPESVFLAPKLYALKCL
1069
KNA-08975           METRGKKRIKKHGGQLVFDPQRPELTWLVECETTCAQCGADTFSPETVFLAPKLYALKCL
1069
SAdV-F/SAdV-18      METRGKKRIKKHGGQLVFDPQHPELTWLVECETTCAQCGADAYSPESVFLAPKLYALKCL
1080
SADV-F/SAdV-17      METRGKKRIKKHGGQLVFDPQRPELTWLVECETTCAQCGADAYSPESVFLAPKLYALKCL
1078
HAdV-F/HAdV-40      METRGKKRIKKHGGNLVFDPKHPELAWLVECETVCAQCGADAYSPESVFLAPKLYALKCL
1076
HAdV-F/HAdV-41      METRGKKRIKKHGGSLVFDPKNPELTWLVECETTCAQCGANAYSPESVFLAPKLYALKCL
1075
                    **************.*****:.***:*******.******:::***:*************

KNA-S6              QCPACGHVSKGKLRAKGHAAEALSYNLMLRCYLADSQGEDARFHTSRMSLKRTLASAQPG
1129
KNA-08975           QCPACGHVSKGKLRAKGHAAEALSYDLMLRCYLADSQGEDARFHTSRMSLKRTLASAQPG
1129
SAdV-F/SAdV-18      HCPACGHVSKGKLRAKGHAAEALSYDLMLKCYLADSQGEDARFHTSRMSLKRTLASAQPG
1140
SADV-F/SAdV-17      QCPACGHVSKGKLRAKGHAAEALSYDLMLKCYLADSQGEDARFHTSRMSLKRTLASAQPG
1138
HAdV-F/HAdV-40      RCPSCQQISKGKLRAKGHAAETLNYDLMLKCYLADFQGEDARFHTSRMSLKRTLASAQPG
1136
HAdV-F/HAdV-41      YCPSCQHISKGKLRAKGHAAEALSYELMLKCYLADSQGEDARFHTSRMSLKRTLASAQPG
1135
                     **:* ::*************:*.*:***:***** ************************

KNA-S6              AHPFTVTETTLTRTLRPWRDVTLAPLDAHRLVPYSQSRPNPRNQEVCWI--- 1178
KNA-08975           AHPFTVTETTLTRTLRPWRDVTLAPLDAHRLVPYSQSRPNPRNQEVCWI--- 1178
SAdV-F/SAdV-18      AHPFTVTETTLTRTLRPWKDVTLASLDAHRLVPYSQSRPNPRNQEVCWIEMP 1192
SADV-F/SAdV-17      AHPFTVTETTLTRTLRPWRDVTLAPLDAHRLVPYSQSRPNPRNQEVCWIEMP 1190
HAdV-F/HAdV-40      ARPFTVTETNLTRTLRPWKDITLAPLDAHRLVPYSQSRPNPRNQEVCWIEMP 1188
HAdV-F/HAdV-41      AHPFTVTETTLTRTLRPWKDITLAPLDAHRLVPYSQSRPNPRNQEVCWIEMP 1187
```

```
                              *:*******.********:*:***.************************
```

Figure S3. Multiple alignment of the nearly complete deduced amino acid (aa) sequences of the putative DNA-dependent DNA polymerase (Pol) of simian adenovirus (SAdV) strains KNA-S6 and KNA-08975 with cognate sequences of SADV-F/SAdV-17 strain B-105 (GenBank accession number KP329566), SAdV-F/SAdV-18 strain C676 (FJ025931), Human AdV-F (HAdV-F)/HAdV-40 isolate Dugan (L19443), and HAdV-F/HAdV-41 isolate Tak (DQ315364). Numbers to the right indicate the positions of the aa for respective AdV strains.

```
KNA-S6                        MATPSMMPQWSYMHIAGQDASEYLSPGLVQFARATDTYFSLGNKFRNPTVAPTHDVTTDR
60
HAdV-F/HAdV-40                MATPSMMPQWSYMHIAGQDASEYLSPGLVQFARATDTYFSLGNKFRNPTVAPTHDVTTDR
60
SAdV-F/SAdV-17                MATPSMMPQWSYMHIAGQDASEYLSPGLVQFARATDTYFSLGNKFRNPTVAPTHDVTTDR
60
KNA-08975                     MATPSMMPQWSYMHIAGQDASEYLSPGLVQFARATDTYFSLGNKFRNPTVAPTHDVTTDR
60
SAdV-F/SAdV-18                MATPSMMPQWSYMHIAGQDASEYLSPGLVQFARATDTYFSLGNKFRNPTVAPTHDVTTDR
60
HAdV-F/HAdV-41                MATPSMMPQWSYMHIAGQDASEYLSPGLVQFARATDTYFSLGNKFRNPTVAPTHDVTTDR
60
                              ************************************************************

KNA-S6                        SQRLTLRFVPVDREDTAYSYKVRFTLAVGDNRVLDMASTYFDIRGVLDRGPSFKPYSGTA
120
HAdV-F/HAdV-40                SQRLTLRFVPVDREETAYSYKVRFTLAVGDNRVLDMASTYFDIRGVLDRGPSFKPYSGTA
120
SAdV-F/SAdV-17                SQRLTLRFVPVDREDTAYSYKVRFTLAVGDNRVLDMASTYFDIRGVLDRGPSFKPYSGTA
120
KNA-08975                     SQRLTLRFVPVDREDTAYSYKVRFTLAVGDNRVLDMASTYFDIRGVLDRGPSFKPYSGTA
120
SAdV-F/SAdV-18                SQRLTLRFVPVDREDTAYSYKVRFTLAVGDNRVLDMASTYFDIRGVLDRGPSFKPYSGTA
120
HAdV-F/HAdV-41                SQRLTLRFVPVDREDTAYSYKVRFTLAVGDNRVLDMASTYFDIRGVLDRGPSFKPYSGTA
120
                              **************:*********************************************

KNA-S6                        YNSLAPKGAPNPSQWTNSVTNTKTSSFGQAPFIGEKITNE-GVQVGTDT--SGGDVFADK
177
HAdV-F/HAdV-40                YNSLAPKGAPNPSQWTN---QNKTNSFGQAPYIGQKITNQ-GVQVGSDS--NNRDVFADK
174
SAdV-F/SAdV-17                YNSLAPKNAPNACQWTSTTNGNKTNTFAQAPFIGLSITKD-GVQVGVDTSQTQQAVYADK
179
KNA-08975                     YNSLAPKGAPNPSEWKG--TDSKVNVRGQAPFFSTSISKD-GIQVGTDTSTPTQAIYADK
177
SAdV-F/SAdV-18                YNSLAPKGAPNPSEWKG--SDNKISVRGQAPFFSTSITKD-GIQVATDTSS--GAVYAKK
175
HAdV-F/HAdV-41                YNSLAPKTAPNPCEWK---DNNKIKVRGQAPFIGTNINKDNGIQIGTDTTN--QPIYADK
175
                              ******* ***..:*.     .* .  .***::. .*.:: *:*:. *:      ::*.*

KNA-S6                        TFQPEPQVGQTQWNIDPTQN--AAGRILKKTTPMQPCYGSYARPTNEQGGQAKLVVNG-G
234
HAdV-F/HAdV-40                TYQPEPQVGQTQWNINPMQN--AAGRILKQTTPMQPCYGSYARPTNEKGGQAKLVKNDDN
232
SAdV-F/SAdV-17                SFQPEPQVGESQWNSNPTTN--AAGRVLKPTTAMLPCYGSYAYPTNEKGG----------
227
KNA-08975                     TYQPEPQVGQEQWNSEAGDNDKVAGRVLKDSTPMFPCYGSYAKPTNEHGGQCTNSTVDLQ
237
SAdV-F/SAdV-18                EYQPEPQVGQEQWNSEASDSDKVAGRILKDTTPMFPCYGSYAKPTNEQGGQGTN-TVDLQ
234
HAdV-F/HAdV-41                TYQPEPQVGQTQWNSEVGAAQKVAGRVLKDTTPMLPCYGSYAKPTNEKGGQASLITNGTD
235
                               :*******: *** :      .***:** :*.* ******* ****:**

KNA-S6                        QTQTTDVSLNFFTTASESSSFTPKVVLYGEDVNLEAPDTHLVFKPDTND--TSAEILLGQ
292
```

```
HAdV-F/HAdV-40       QTTTTNVGLNFFTTATETANFSPKVVLYSEDVNLEAPDTHLVFKPDVNG--TSAELLLGQ
290
SAdV-F/SAdV-17       QTKTQDGVDLKFFTTAGDNANQPKVVLYSEDVNLEAPDTHIVFKPDPEA--TNSEAMLTQ
285
KNA-08975            FFASSGG-----------SNNTPKAVLYAEDVNIEAPDTHLVYKPSVTQGTTSSQDLLTQ
286
SAdV-F/SAdV-18       FFASSS------------ATSTPKAVLYAEDVAIEAPDTHLVYKPAVTTTTTSSQDLLTQ
282
HAdV-F/HAdV-41       QTLTSDVNLQFFALPS--TPNEPKAVLYAENVSIEAPDTHLVYKPDVAQGTISSADLLTQ
293
                        : .                **.***.*:* :******:*:**       .:  :* *

KNA-S6               QASPNRPNYIGFRDNFIGLMYYNSTGNMGVLAGQASQLNAVVDLQDRNTELSYQLMLDAL
352
HAdV-F/HAdV-40       QAAPNRPNYIGFRDNFIGLMYYNSTGNMGVLAGQASQLNAVVDLQDRNTELSYQLMLDAL
350
SAdV-F/SAdV-17       QAAPNRPNYVAFRDNFIGLMYYNSTGNMGVLAGQASQLNAVVDLQDRNTELSYQLMLDAL
345
KNA-08975            QAAPNRPNYIGFRDNFIGLMYYNSTGNMGVLAGQASQLNAVVDLQDRNTELSYQLMLDAL
346
SAdV-F/SAdV-18       QAAPNRPNYIGFRDNFIGLMYYNSTGNMGVLAGQASQLNAVVDLQDRNTELSYQLMLDAL
342
HAdV-F/HAdV-41       QAAPNRPNYIGFRDNFIGLMYYNSTGNMGVLAGQASQLNAVVDLQDRNTELSYQLMLDAL
353
                     **:******:.*************************************************

KNA-S6               GDRSRYFSMWNQAVDSYDPDVRIIENHGVEDELPNYCFPLNGQGIANSYQGVKNGSGSSW
412
HAdV-F/HAdV-40       GDRSRYFSMWNQAVDSYDPDVRIIENHGVEDELPNYCFPLNGQGISNSYQGVKTDNGTNW
410
SAdV-F/SAdV-17       GDRSRYFSMWNQAVDSYDPDVRIIENHGVEDELPNYCFPLSGMGISNTYKGLQS-NGNGG
404
KNA-08975            GDRSRYFSMWNPAVDSYDPDVRTIENHGEEDELPNYCFPLGGSLVTETYSPLKLQNGGSD
406
SAdV-F/SAdV-18       GDRSRYFSMWNQAVDSYDPDVRIIENHGVEDELPNYCFPLGGSLVTETYTGLSPQNG-SN
401
HAdV-F/HAdV-41       GDRSRYFSMWNQAVDSYDPDVRIIENHGVEDELPNYCFPLGGSAATDTYSGIKANGQTWT
413
                     *********** ********** ***** ***********.*   :::*  :.  .


KNA-S6               SKDD---TNVSTNNEISIGNVFAMEINLAANLWRSFLYSNVALYLPDAYKLTPDNITLPD
469
HAdV-F/HAdV-40       SQNN---TDVSSNNEISIGNVFAMEINLAANLWRSFLYSNVALYLPDSYKITPDNITLPD
467
SAdV-F/SAdV-17       FQAD---NTASETNEIGIGNIFAFEINIAANLWRSFLYSNVALYLPDAYKYTPDNITLPD
461
KNA-08975            SWTQDSDTYATRGVEIGSGNMFAMEINLAANLWRIFLYSNVALYLPDAYKLTPDNITLPE
466
SAdV-F/SAdV-18       TWTTDSTTYATRGVEIGSGNMFAMEINLAANLWRSFLYSNVALYLPDEYKLTPDNITLPD
461
HAdV-F/HAdV-41       ADDN----YADRGAEIESGNIFAMEINLAANLWRSFLYSNVALYLPDSYKITPDNITLPE
469
                              .    **  **:**:***:****** ************ ** ********:

KNA-S6               NKNTYDYMNGRVAAPSSLDTYVNIGARWSPDPMDNVNPFNHHRNAGLRYRSMLLGNGRYV
529
HAdV-F/HAdV-40       NKNTYAYMNGRVAVPSALDTYVNIGARWSPDPMDNVNPFNHHRNAGLRYRSMLLGNGRYV
527
SAdV-F/SAdV-17       NKNTYDYINGRVTAPTALDTYVNIGARWSPDPMDNVNPFNHHRNAGLRYRSMLLGNGRYV
521
KNA-08975            NKNTYDYMNGRVAAPSSLDIYVNIGARWSPDPMDNVNPFNHHRNAGLRYRSMLLGNGRYV
526
SAdV-F/SAdV-18       NKNTYDYMNGRVAAPSSLDTYVNIGARWSPDPMDNVNPFNHHRNAGLRYRSMLLGNGRYV
521
HAdV-F/HAdV-41       NKNTYAYMNGRVAVPSALDTYVNIGARWSPDPMDNVNPFNHHRNAGLRYRSMLLGNGRYV
529
                     ***** *:****:.*::** ****************************************
```

```
KNA-S6              PFHIQVPQKFFAIKNLLLLPGSYTYEWNFRKDVNMILQSSLGNDLRVDGASVKFDSINLY
589
HAdV-F/HAdV-40      PFHIQVPQKFFAIKNLLLLPGSYTYEWNFRKDVNMILQSSLGNDLRVDGASVRFDSINLY
587
SAdV-F/SAdV-17      PFHIQVPQKFFAIKNLLLLPGSYTYEWNFRKDVNMILQSSLGNDLRVDGASVRFDSINLY
581
KNA-08975           PFHIQVPQKFFAIKNLLLLPGSYTYEWNFRKDVNMILQSSLGNDLRVDGASVRFDSINLY
586
SAdV-F/SAdV-18      PFHIQVPQKFFAIKNLLLLPGSYTYEWNFRKDVNMILQSSLGNDLRVDGASVRFDSINLY
581
HAdV-F/HAdV-41      PFHIQVPQKFFAIKNLLLLPGSYTYEWNFRKDVNMILQSSLGNDLRVDGASVRFDSINLY
589
                    ****************************************************:*******

KNA-S6              ANFFPMAHNTASTLEAMLRNDTNDQSFNDYLCAANMLYPIPANATSVPISIPSRNWAAFR
649
HAdV-F/HAdV-40      ANFFPMAHNTASTLEAMLRNDTNDQSFNDYLCAANMLYPIPANATSVPISIPSRNWAAFR
647
SAdV-F/SAdV-17      ANFFPMAHNTASTLEAMLRNDTNDQSFNDYLCAANMLYPIPANATSVPISIPSRNWAAFR
641
KNA-08975           ANFFPMAHNTASTLEAMLRNDTNDQSFNDYLCAANMLYPIPANATSVPISIPSRNWAAFR
646
SAdV-F/SAdV-18      ANFFPMAHNTASTLEAMLRNDTNDQSFNDYLCAANMLYPIPANATSVPISIPSRNWAAFR
641
HAdV-F/HAdV-41      ANFFPMAHNTASTLEAMLRNDTNDQSFNDYLCAANMLYPIPSNATSVPISIPSRNWAAFR
649
                    *****************************************:******************

KNA-S6              GWSFTRLKTKETPSLGSGFDPYFTYSGSIPYLDGTFYLNHTFKKVSVMFDSSVSWPGNDR
709
HAdV-F/HAdV-40      GWSFTRLKTKETPSLGSGFDPYFTYSGSVPYLDGTFYLNHTFKKVSVMFDSSVSWPGNDR
707
SAdV-F/SAdV-17      GWSFTRLKTKETPSLGSGFDPYFTYSGSIPYLDGTFYLNHTFKKVSIMFDSSVSWPGNDR
701
KNA-08975           GWSFTRLKTRETPSLGSGFDPYFTYSGSIPYLDGTFYLNHTFKKVSIMFDSSVSWPGNDR
706
SAdV-F/SAdV-18      GWSFTRLKTKETPSLGSGFDPYFTYSGSIPYLDGTFYLNHTFKKVSIMFDSSVSWPGNDR
701
HAdV-F/HAdV-41      GWSFTRLKTKETPSLGSGFDPYFTYSGSVPYLDGTFYLNHTFKKVSIMFDSSVSWPGNDR
709
                    *********:******************:*****************:*************

KNA-S6              LLTPNEFEIKRTVDGEGYNVAQCNMTKDWFLIQMLSHYNIGYQGFYVPEGYKDRMYSFFR
769
HAdV-F/HAdV-40      LLTPNEFEIKRTVDGEGYNVAQCNMTKDWFLIQMLSHYNIGYQGFHVPESYKDRMYSFFR
767
SAdV-F/SAdV-17      LLTPNEFEIKRTVDGEGYNVAQCNMTKDWFLIQMLSHYNIGYQGFYVPEGYKDRMYSFFR
761
KNA-08975           LLTPNEFEIKRTVDGEGYNVAQCNMTKDWFLIQMLSHYNIGYQGFYVPEGYKDRMYSFFR
766
SAdV-F/SAdV-18      LLTPNEFEIKRTVDGEGYNVAQCNMTKDWFLIQMLSHYNIGYQGFYVPEGYKDRMYSFFR
761
HAdV-F/HAdV-41      LLTPNEFEIKRTVDGEGYNVAQCNMTKDWFLIQMLSHYNIGYQGFYVPESYKDRMYSFFR
769
                    *********************************************:***.**********

KNA-S6              NFQPMSRQVVDTTSYSDYKNVTLPFQHNNSGFVGYMGPTMREGQAYPANYPYPLIGKTAV
829
HAdV-F/HAdV-40      NFQPMSRQVVDTTTYTEYQNVTLPFQHNNSGFVGYMGPAIREGQAYPANYPYPLIGQTAV
827
SAdV-F/SAdV-17      NFQPMSRQVVDSVNYANYKEVKLPFQHNNSGFVGYMGPTMREGQAYPANYPYPLIGKTAV
821
KNA-08975           NFQPMSRQVVDTTTYSDYQNVTLPFQHNNSGFVGYMGPTMREGQAYPANYPYPLIGKTAV
826
SAdV-F/SAdV-18      NFQPMSRQVVDTTTYTDYKNVTLPFQHNNSGFVGYMGPTMREGQAYPANYPYPLIGKTAV
821
HAdV-F/HAdV-41      NFQPMSRQVVNTTTYKEYQNVTLPFQHNNSGFVGYMGPTMREGQAYPANYPYPLIGQTAV
829
                    **********::..* :*::*.****************::****************:***
```

```
KNA-S6              PSLTQKKFLCDRTMWRIPFSSNFMSMGALTDLGQNMLYANSAHALDMTFEVDPMDEPTLL
889
HAdV-F/HAdV-40      PSLTQKKFLCDRTMWRIPFSSNFMSMGALTDLGQNMLYANSAHALDMTFEVDPMDEPTLL
887
SAdV-F/SAdV-17      DSLTQKKFLCDRTMWRIPFSSNFMSMGALTDLGQNMLYANSAHALDMTFEVDPMDEPTLL
881
KNA-08975           PSLTQKKFLCDRTMWRIPFSSNFMSMGALTDLGQNMLYANSAHALDMTFEVDPMDEPTLL
886
SAdV-F/SAdV-18      PSLTQKKFLCDRTMWRIPFSSNFMSMGALTDLGQNMLYANSAHALDMTFEVDPMDEPTLL
881
HAdV-F/HAdV-41      PSLTQKKFLCDRTMWRIPFSSNFMSMGALTDLGQNMLYANSAHALDMTFEVDPMDEPTLL
889
                     ***********************************************************


KNA-S6              YVLFEVFDVVRIHQPHRGVIEAVYLRTPFSAGNATT 925
HAdV-F/HAdV-40      YVLFEVFDVVRIHQPHRGVIEAVYLRTPFSAGNATT 923
SAdV-F/SAdV-17      YVLFEVFDVVRIHQPHRGVIEAVYLRTPFSAGNATT 917
KNA-08975           YVLFEVFDVVRIHQPHRGVIEAVYLRTPFSAGNATT 922
SAdV-F/SAdV-18      YVLFEVFDVVRIHQPHRGVIEAVYLRTPFSAGNATT 917
HAdV-F/HAdV-41      YVLFEVFDVVRIHQPHRGVIEAVYLRTPFSAGNATT 925
                    ************************************
```

Figure S4. Multiple alignment of the complete deduced amino acid (aa) sequences of the putative hexons of simian adenovirus (SAdV) strains KNA-S6 and KNA-08975 with those of SADV-F/SAdV-17 strain B-105 (GenBank accession number KP329566), SAdV-F/SAdV-18 strain C676 (FJ025931), Human AdV-F (HAdV-F)/HAdV-40 isolate Dugan (L19443), and HAdV-F/HAdV-41 isolate Tak (DQ315364). The region of the putative hexon (genetically divergent between the hexons of SAdV-17, -18, and HAdV-F) that formed the basis of classification of the AdV strains from African green monkeys into group-I and group-II viruses is highlighted with blue. Numbers to the right indicate the positions of the aa for respective AdV strains.

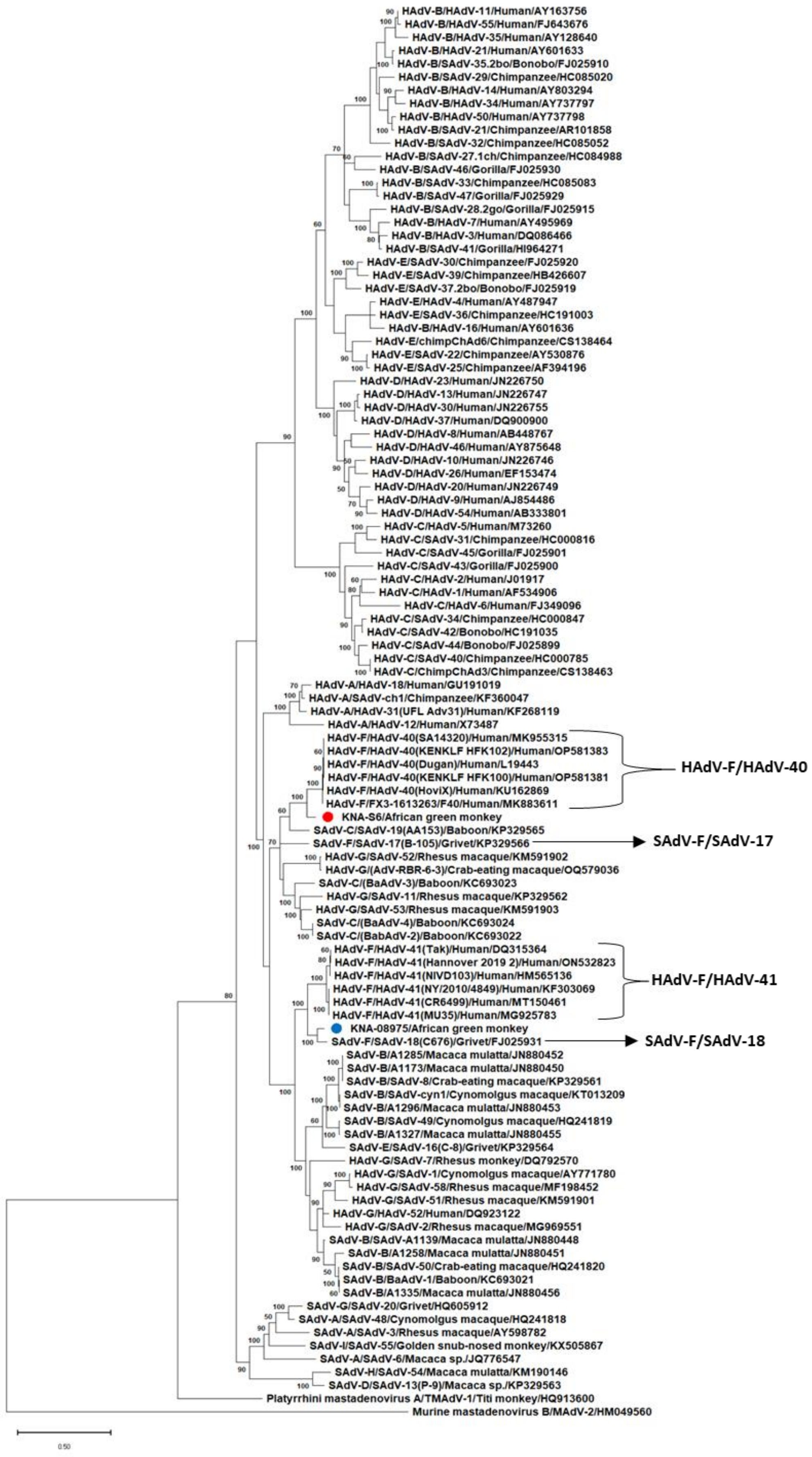

Figure S5. Expanded version of figure 4.

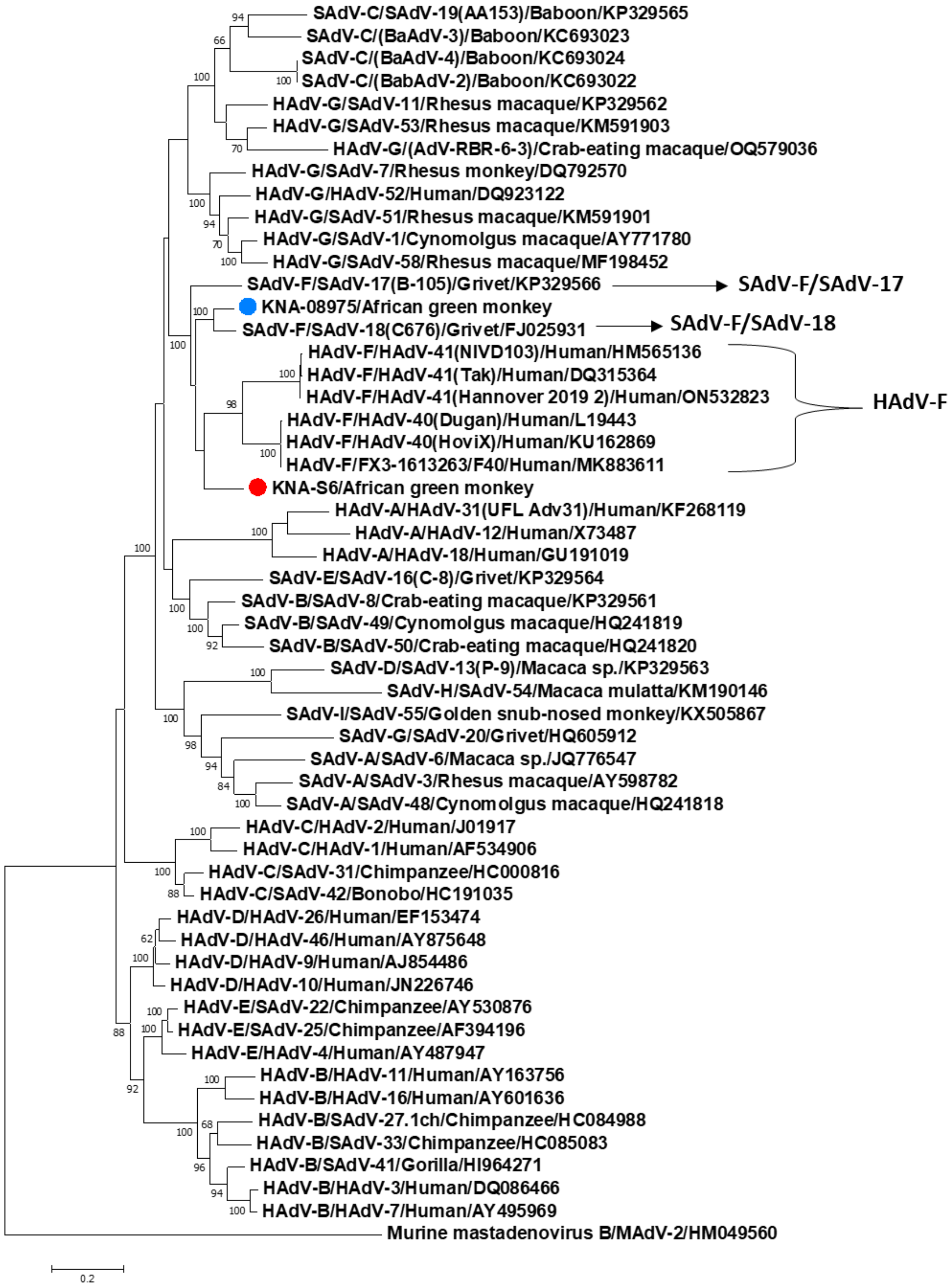

Figure S6. Phylogenetic analysis of the complete hexon coding sequences of simian adenovirus (SAdV) strains KNA-S6 and KNA-08975 (shown with red and blue circles, respectively) with those of human adenoviruses (HAdVs) and other SAdVs. The tree was constructed using the maximum likelihood (ML) method with the Hasegawa-Kishino-Yano model + G and 1000 bootstrap replicates. Similar clustering patterns were observed when the ML analysis was repeated using other mathematical models. *Murine mastadenovirus B*/MAdV-2/HM049560 was used as the outgroup sequence. Bootstrap values < 60% are not shown. Scale bar, 0.2 substitutions per nucleotide.

```
                    210
KNA-S6              NFRLGWDPVTKLVMPGVYTNEAFHPDIVLLPGCGVDFTQSRLSNLLGIRKRMPFQEGFQI
SAdV-F/SAdV-17      NFRLGWDPVTKLVMPGVYTNEAFHPDIVLLPGCGVDFTQSRLSNLLGIRKRMPFQAGFQI
KNA-08975           NFRLGWDPVTKLVMPGVYTNEAFHPDIVLLPGCGVDFTQSRLSNLLGIRKRMPFQEGFQI
SAdV-F/SAdV-18      NFRLGWDPVTKLVMPGVYTNEAFHPDIVLLPGCGVDFTQSRLSNLLGIRKRMPFQAGFQI
HAdV-F/HAdV-40      NFRLGWDPVTKLVMPGVYTNEAFHPDIVLLPGCGVDFTQSRLNNLLGIRKRMPFQKGFQI
HAdV-F/HAdV-41      NFRLGWDPVTKLVMPGVYTNEAFHPDIVLLPGCGVDFTQSRLSNLLGIRKRLPFQEGFQI
                    ******************************************.********:*** ****

KNA-S6              MYNDLEGGNIPALLDVAKYEASITEAQQQGKEIRGDTFAVSPQDLVIEPVANDSKNRSYN
SAdV-F/SAdV-17      MYEDLEGGNIPALLDVAKYEASITQAQQQGKEIRGDTFAVSPQDLDIEPVEKDSKNRSYN
KNA-08975           MYEDLEGGNIPALLDVAKYEASIQQAREQGQEIRGDNFTVIPRDVEIVPVEQDSKGRSYN
SAdV-F/SAdV-18      MYEDLEGGNIPALLDVAKYEASIQKAREQGQEIRGDNFTVIPRDVEIVPVEKDSKDRSYN
HAdV-F/HAdV-40      MYEDLEGGNIPALLDVEKYEASIKEA----QEIRGADFKPNPQDLEIVPVEKDSKERSYN
HAdV-F/HAdV-41      MYEDLEGGNIPALLDVAKYEASIQKAKEEGKEIGDDTFATRPQDLVIEPVAKDSKNRSYN
                    **:************* ****** :*    :** .  *   *:*: * ** :*** ****

KNA-S6              LLPDDKNNTAYRSWFLAYNYGDPEKGVRSWTLLTTTDVTCGPQQVYWSLPDMMQDPVTFR
SAdV-F/SAdV-17      LLPDDKNNTAYRSWFLAYNYGDPEKGVRSWTLLTTTDVTCGSQQVYWSLPDMMQDPVTFR
KNA-08975           LIPTDKTNTEYRSWFLAYNYGDPEKGVRSWTLLTTTDVTCGSQQVYWSLPDMMQDPVTFR
SAdV-F/SAdV-18      LLPGDQTNTAYRSWFLAYNYGDPEKGVRSWTLLTTTDVTCGSQQVYWSLPDMMQDPVTFR
HAdV-F/HAdV-40      LLEGDKNNTAYRSWFLAYNYGDAEKGVKSWTLLTTTDVTCGSQQVYWSLPDMMQDPVTFR
HAdV-F/HAdV-41      LLPNDQNNTAYRSWFLAYNYGDPKKGVQSWTLLTTADVTCGSQQVYWSLPDMMQDPVTFR
                    *:  *:.** ************.:***:*******:*****.******************

KNA-S6              PSSQVSNYPVVGVELLPVHAKSFYNEQAVYSQLIRQSTALTHVFNRFPENQILVRPPAPT
SAdV-F/SAdV-17      PSSQVSNYPVVGVELLPVHAKSFYNEQAVYSQLIRQSTALTHVFNRFPENQILVRPPAPT
KNA-08975           PSSQVSNYPVVGVELLPVHAKSFYNEQAVYSQLIRQSTALTHVFNRFPENQILVRPPAPT
SAdV-F/SAdV-18      PSSQVSNYPVVGVELLPVHAKSFYNEQAVYSQLIRQSTALTHVFNRFPENQILVRPPAPT
HAdV-F/HAdV-40      PSTQVSNYPVVGVELLPVHAKSFYNEQAVYSQLIRQSTALTHIFNRFPENQILVRPPAPT
HAdV-F/HAdV-41      PSTQVSNYPVVGVELLPVHAKSFYNEQAVYSQLIRQSTALTHVFNRFPENQILVRPPAPT
                    **:***************************************:*****************
                                                                              508
KNA-S6              ITTVSENVPALTDHGTLPLRSSISGVQRVTITDARRRTCPYVHKALGIVAPKVLSSRTF
SAdV-F/SAdV-17      ITTVSENVPALTDHGTLPLRSSISGVQRVTITDARRRTCPYVHKALGIVAPKVLSSRTF
KNA-08975           ITTVSENVPALTDHGTLPLRSSISGVQRVTITDARRRTCPYVHKALGIVAPKVLSSRTF
SAdV-F/SAdV-18      ITTVSENVPALTDHGTLPLRSSISGVQRVTITDARRRTCPYVHKALGIVAPKVLSSRTF
HAdV-F/HAdV-40      ITTVSENVPALTDHGTLPLRSSISGVQRVTITDARRRTCPYVHKALGIVAPKVLSSRTF
HAdV-F/HAdV-41      ITTVSENVPALTDHGTLPLRSSISGVQRVTITDARRRTCPYVHKALGIVAPKVLSSRTF
                    ***********************************************************
```

Figure S7. Multiple alignment of the partial deduced amino acid (aa) sequences of the putative penton bases of simian adenovirus (SAdV) strains KNA-S6 and KNA-08975 with cognate sequences of SADV-F/SAdV-17 strain B-105 (GenBank accession number KP329566), SAdV-F/SAdV-18 strain C676 (FJ025931), Human AdV-F (HAdV-F)/HAdV-40 isolate Dugan (L19443), and HAdV-F/HAdV-41 isolate Tak (DQ315364). The putative 'RGD' motif is highlighted with green and was absent in HAdV-F strains. The aa numbers shown here correspond to those of the complete deduced aa sequence of penton base of SAdV-F/SAdV-18.

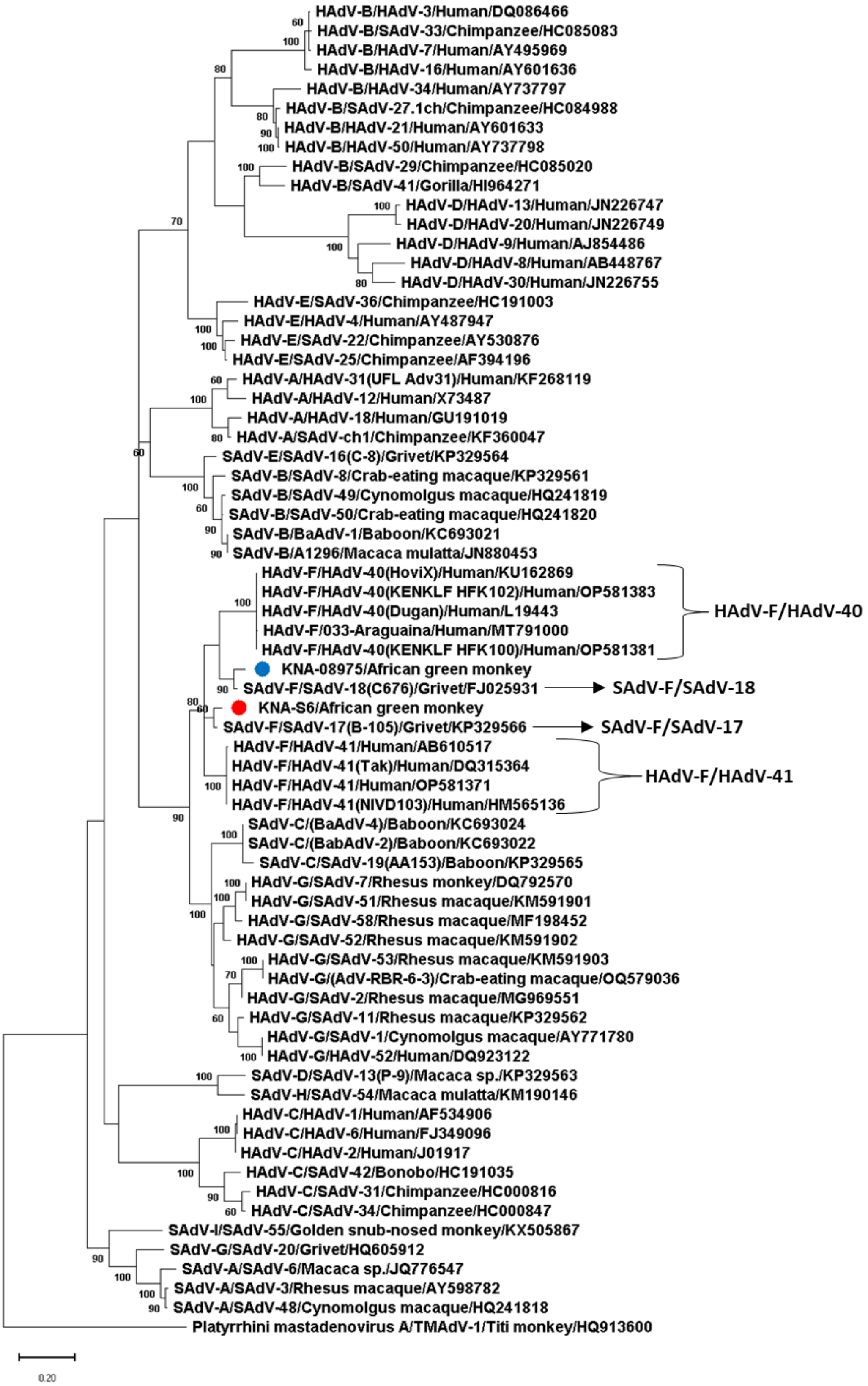

Figure S8. Expanded version of figure 5.

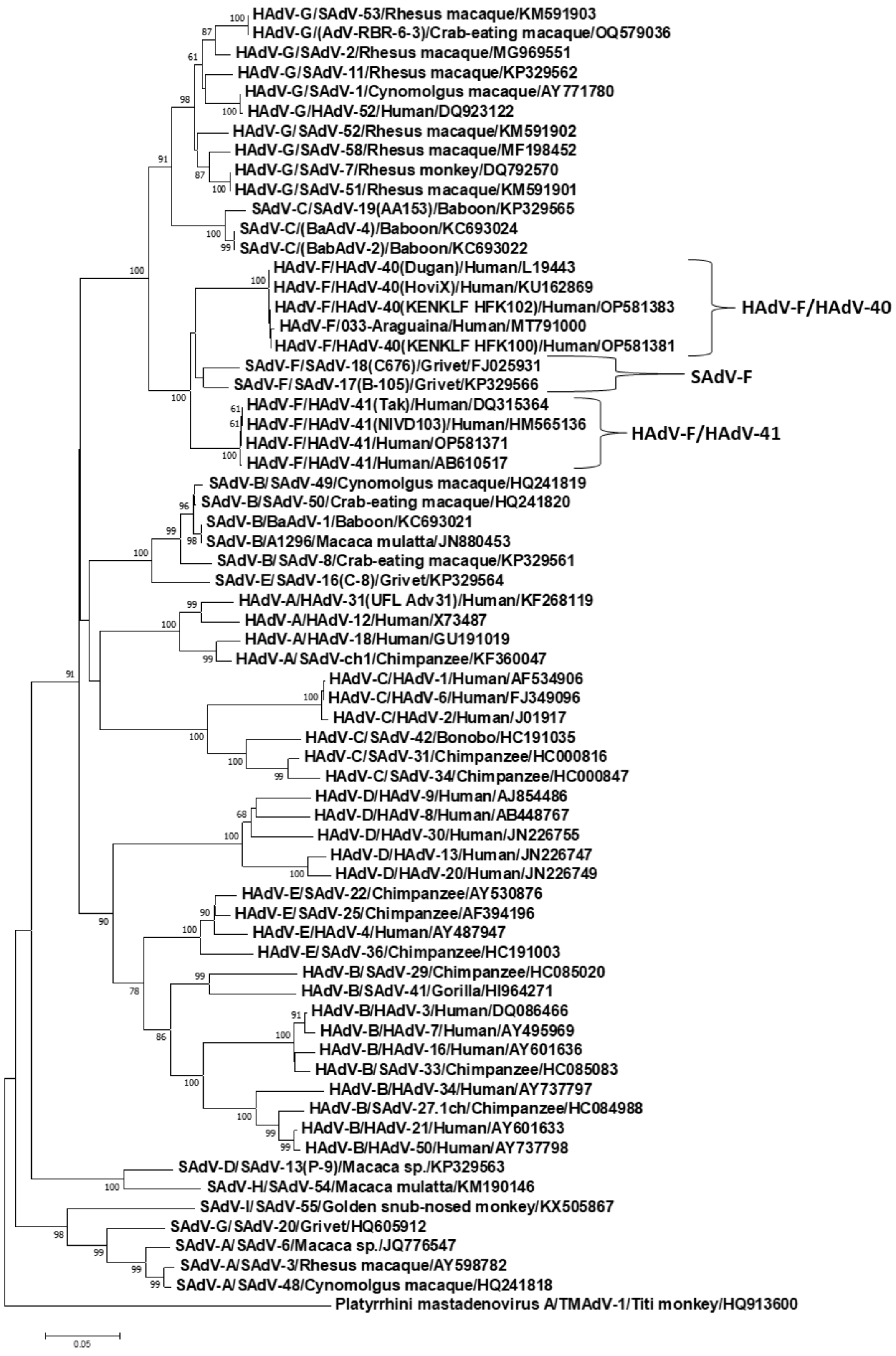

Figure S9. Phylogenetic analysis of the complete deduced amino acid (aa) sequences of the putative penton bases of simian adenoviruses (SAdVs) and human adenoviruses (HAdVs). The phylogenetic analysis was performed as described under 'Materials and Methods' section. Bootstrap values < 60% are not shown. Scale bar, 0.05 substitutions per aa residue.
